# Comparative Analysis of DNA Replication Timing Reveals Conserved Large-Scale Chromosomal Architecture

**DOI:** 10.1371/journal.pgen.1001011

**Published:** 2010-07-01

**Authors:** Eitan Yaffe, Shlomit Farkash-Amar, Andreas Polten, Zohar Yakhini, Amos Tanay, Itamar Simon

**Affiliations:** 1 Department of Computer Science and Applied Mathematics, Weizmann Institute of Science, Rehovot, Israel; 2 Department of Microbiology and Molecular Genetics, the Institute for Medical Research – Israel-Canada, the Hebrew University-Hadassah Medical School, Jerusalem, Israel; 3 Agilent Technologies, Waldbronn, Germany; 4 Department of Computer Sciences, Technion – Israel Institute of Technology, Haifa, Israel; 5 Agilent Laboratories, Tel-Aviv, Israel; Medical Research Council Human Genetics Unit, United Kingdom

## Abstract

Recent evidence suggests that the timing of DNA replication is coordinated across megabase-scale domains in metazoan genomes, yet the importance of this aspect of genome organization is unclear. Here we show that replication timing is remarkably conserved between human and mouse, uncovering large regions that may have been governed by similar replication dynamics since these species have diverged. This conservation is both tissue-specific and independent of the genomic G+C content conservation. Moreover, we show that time of replication is globally conserved despite numerous large-scale genome rearrangements. We systematically identify rearrangement fusion points and demonstrate that replication time can be locally diverged at these loci. Conversely, rearrangements are shown to be correlated with early replication and physical chromosomal proximity. These results suggest that large chromosomal domains of coordinated replication are shuffled by evolution while conserving the large-scale nuclear architecture of the genome.

## Author Summary

During S-phase of the cell cycle, chromosomal DNA is replicated in a complex process involving the coordinated activity of thousands of replication forks, each of which duplicates a long stretch of DNA. Recent experiments revealed that the genome is replicating as a mosaic of large-scale early and late chromosomal domains and that this high-level domain organization is correlated with genomic properties like gene density and nucleotide composition. We compared genome-wide replication time maps of compatible human and mouse cells and revealed that their organization into replication domains is highly conserved despite the numerous large-scale genome rearrangements separating the two species. Analysis of recent chromosomal interaction data shows that regions with similar time of replication are more frequently interacting with each other than expected. The data also show that evolutionary rearrangements have predominantly occurred between regions that have similar time of replication and higher-than-expected chromosomal proximity. Our data suggests that the genome, while being continuously rearranged by evolution, maintains a conserved domain organization. Whether this conservation is driven by selection, or is a consequence of the rearrangement process itself, can be resolved by enhancing the comparative approach proposed here.

## Introduction

Mammalian genomes are complex and heterogeneous entities, consisting of many thousands of functional elements that are packed into chromosomes and organized in nuclear space. Our understanding of the global implications of genome organization, its effect on gene regulation and its evolutionary consequences is still quite limited. Recent advances in epigenomic profiling have begun to uncover large-scale genomic domains that are marked with specific histone modifications [1]–[5], interact with important nuclear landmarks [6] or replicate as units at specific times during S phase [7]–[14]. Data on inter chromosomal interactions hint as to how such large scale domains may be organized in the three-dimensional nucleus structure [15]. Yet the origin of large-scale genome organization is unclear: How does the genome self-organize into domains? How are these domains exploited for regulation and how can the cell propagate them to daughter cells? From an evolutionary perspective, the extent to which the genome's domain organization is conserved is unclear, as are the evolutionary mechanisms that contribute to such conservation [16], [17]. Even if domains are conserved, the origin of such conservation may have several explanations. If domains are functionally important, for example as scaffolds for gene clusters [18], we may expect genome rearrangements that break them to be selected against. On the other hand, if genome rearrangements are enriched at particular hotspots [19], [20], or are affected by various epigenetic factors, the genome may conserve domains with low rates of rearrangements without selection.

Genomic replication domains were shown to exhibit a particularly robust large-scale behavior. Domains of tens of kilobases to megabases collectively replicate at particular timings during S-phase in mice [7], [8], [21], human [9], [12]–[14], [22], [23] and flies [10], [11]. Such modular behavior was suggested to be driven by the coordinated firing of a large number of spatially clustered origins of replication. Recently, studies in mouse and human cells reveals that approximately one third of the genome changes its ToR between tissues [8], [21], [23]. DNA replication timing was shown to be highly correlated with other genomic features, most notably the regional G+C content but also gene density, gene expression, open chromatin and mutability (reviewed in [24]). Genomic replication domains therefore naturally describe an important type of large-scale genomic organization and are ideal markers for studying such organization from an evolutionary perspective.

In this work, we measure and compare the time of replication of the human and mouse genomes. We use the data to test the correlation between the divergence of large-scale chromosome structure and the divergence of replication timing. We find that while chromosome structure is constantly being challenged by evolution, the genome's time of replication is remarkably conserved. Our analysis of the correlation between genome rearrangements, time of replication and chromosomal conformation suggests that the evolution of chromosome architecture may be confined by the static and dynamic organization of the genome in the nucleus. These results put some of the open questions on chromosome structure and function in a new evolutionary perspective and suggest that additional comparative analysis may be important for their investigation

## Results

We followed the technical approach of Woodfine et al. [13], [14], and quantified the time of replication (ToR) of mammalian genomic replication domains by sorting G1 and S phase cells, and measuring the ratio between their DNA contents using custom design two dye microarray technology (Agilent Technologies; Materials and Methods, Figure S1). We confirmed that the ToR profiles thus derived fit well with mouse ToR profiles generated by us and others using alternative protocols (Farkash-Amar et al. [7] 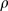 = 0.8 Figure S2, Hiratani et al. [8] 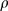 = 0.72). We measured ToR profiles of human fibroblasts (FFT) and lymphoblasts (Molt-4), and of mouse embryonic fibroblasts (MEF) and lymphoblasts (L1210), using ∼105K genomic probes that were designed independently for each species (GEO database, GSE17236). The standardized experimental protocols and downstream computational analysis enabled a rigorous cross-species and cross-tissues comparative ToR analysis. The human and mouse genome differ from each other by a large number of genome rearrangements, but over 90% of the genomes can be mapped into corresponding syntenic regions. We used the human-mouse genome alignment [25]–[28] to project the mouse ToR data onto syntenic human orthologous regions. The comparison of human and mouse ToR was done over large genomic bins (50Kb) and was based on non-orthologous probes, which ensured that our results were not affected by hybridization biases. The use of non orthologous probes can be justified by the fact that ToR changes slowly along the genome, i.e., there is a high degree of autocorrelation of ToR in relatively large genomic domains (up to 4Mb, see Figure S3) and thus two probes from the same region should report the same ToR. The comparative ToR map of the human and mouse genome we computed covers 1.38 gigabases (49% of the human genome).

### ToR is conserved between human and mouse

**Figure 1:**
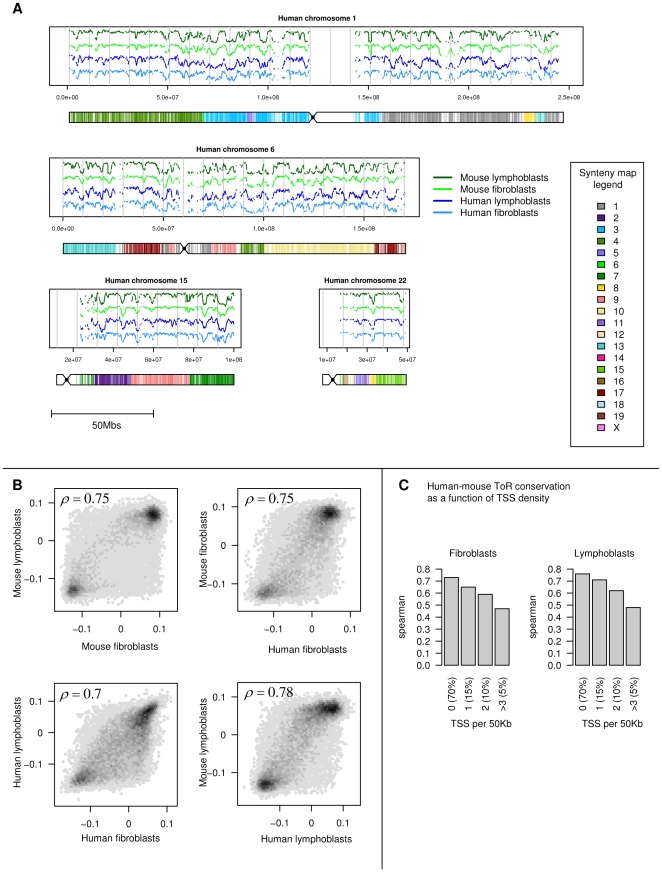
Conservation of time of replication in human and mouse cells. (A) Conservation of the replication profiles. Shown are ToR profiles for human chromosomes 1, 6, 15 and 21 for human fibroblasts (FFT, light blue), human lymphoblasts (MOLT4, dark blue), and the orthologous time of replication profiles in mouse embryonic fibroblasts (MEF, light green), and mouse lymphoblasts (L1210, dark green). Below each chromosome we show the human-mouse synteny map, color coded according to the corresponding mouse chromosomes. (B) Cross-tissue and species correlations. A two-way comparison between the two human and two mouse ToR profiles. Spearman correlation coefficients are specified in each scatter plot. (C) ToR conservation is higher in gene deserts than in gene rich domains. ToR spearman correlation (human versus mouse) as a function of gene density, represented by the number of transcription start sites per 50kb window.

The human and mouse genome sequences differ locally on about 30% of the nucleotides [29]. Furthermore, the two species are separated by hundreds of large-scale genome rearrangement events (such as fusions, translocations and inversions). Despite these differences the correlation between human and mouse ToR is striking. As shown in Figure 1 (see also Figure S4 and Figure S5), the global replication landscape of the human genome matches that of the mapped mouse regions (overall Spearman 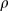 = 0.74 for fibroblasts, and 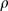 = 0.78 for lymphoblasts, P<10^−100^). The levels of human-mouse ToR correlations are similar to those derived by comparing the two cell types within each species (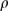 = 0.7 for human, and 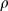 = 0.83 for mouse). This correlation confirms previous observation of ToR conservation which were based on analysis of ToR of genes [7] and expands it to the entire genome. Our estimations of the extent of ToR conservation are higher than those proposed recently [30], probably due to the more careful genome alignment procedure we used here. Our data show that ToR conservation is higher in gene deserts than in gene rich domains (Figure 1C and Figure S6), suggesting that ToR conservation is not a simple consequence of gene expression conservation. Furthermore, the observed conservation is not likely to be a consequence of global sequence conservation, since sequence divergence and ToR divergence are uncorrelated (Spearman 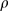 = 0.02). The ToR landscape consists of large-scale domains, as shown before for the mouse genome [7], and we will focus below on the ToR evolution at these scales. We reconfirmed that our 50Kb tiling resolution is capturing most of the large scale ToR structure in the genome by analysis of ToR in one human and one mouse chromosomes that were densely tiled on our arrays (Figure S7).

### Spatial analysis confirms tissue-specific ToR conservation

To systematically characterize genomic domains with evolutionary conserved, diverged or tissue specific time of replication, we used the *spatial clustering algorithm* [31]. The algorithm works in an unsupervised fashion to identify and characterize *spatial clusters*. A spatial cluster is a collection of contiguous genomic regions that display a similar multivariate ToR trend (see Materials and Methods). Data that do not fit any of the clusters is attributed to a default *background cluster*. The algorithm thus identifies frequently recurring patterns in the data, while taking into account the strong spatial coupling between adjacent genomic loci. The algorithm can in theory discover clusters that display ToR conservation or any mixture of diverged ToR trends, allowing the above conclusion on global ToR conservation to be revaluated from a regional perspective. Analysis of the human and mouse aligned maps revealed that 92% of the mapped regions fell into four spatial clusters, all of which display ToR conservation (Figure S8). Interestingly, two of the inferred clusters (representing 25% and 15% of the probes respectively) exhibit distinct tissue-specific replication patterns (clusters 3 and 4 in Figure S8). The ToR difference between the two cell types is conserved between mouse and human, suggesting that tissue-specific ToR is evolutionary conserved. This was directly confirmed by computing profiles of the difference in ToR between fibroblasts and lymphoblasts (for both human and mouse), and measuring the correlation between these profiles across the two species (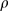 = 0.22, p<10^−292^). Analysis of the four inferred clusters in light of other genomic features confirmed previous observations that late replication regions are significantly poor in genes and transcription and showed additional correlations with genomic features, including increased frequency of interaction with the nuclear lamina and biased distance from the telomere (Figure S9).

### G+C content conservation does not explain ToR conservation

**Figure 2:**
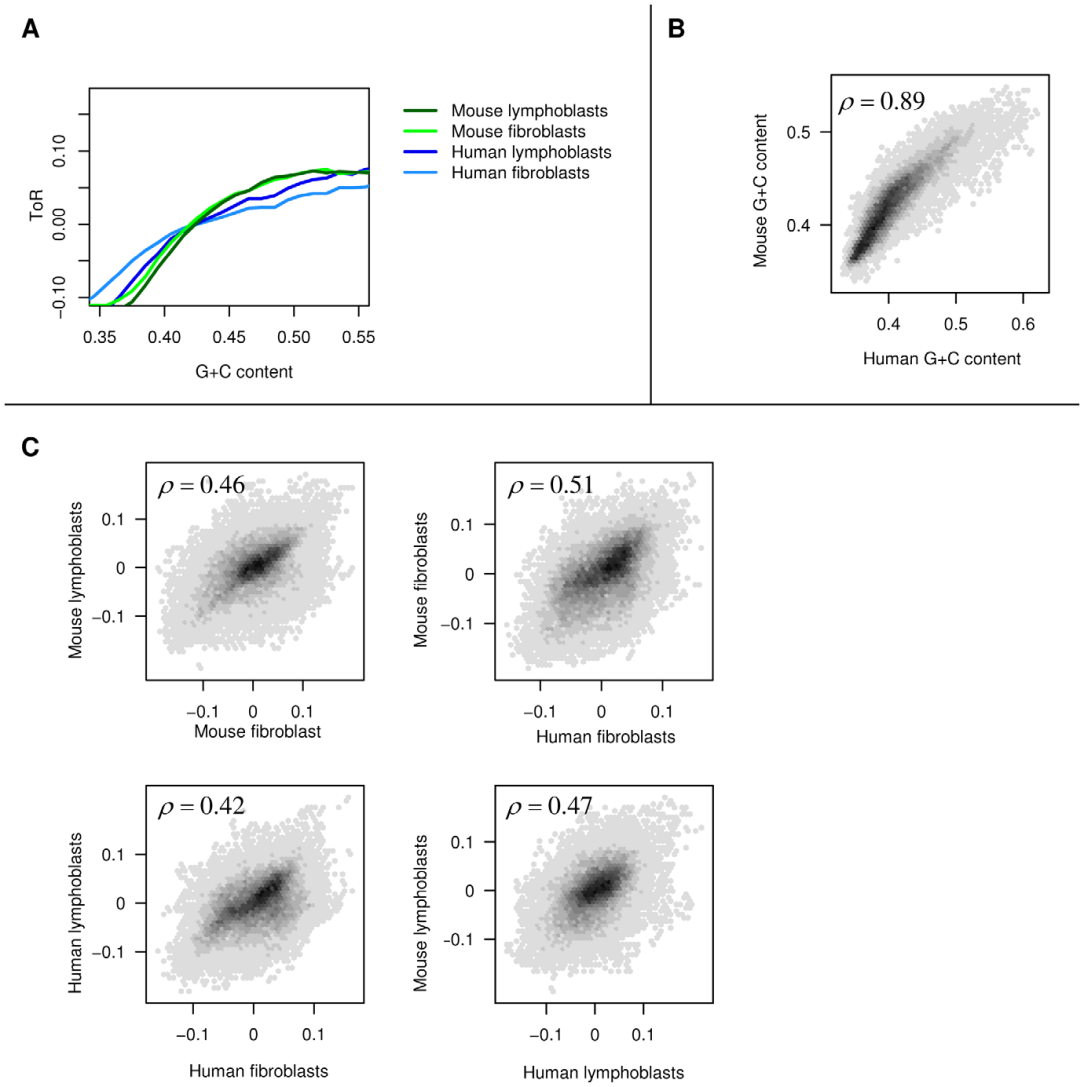
G+C content conservation does not explain ToR conservation. (A) Fitting ToR to G+C content. Shown are moving averages of ToR as a function of G+C content (in 50Kb bins) for the four ToR profiles (using 50 equal-sized G+C content bins ranging between 0.2 and 0.7). (B) Conservation of G+C content between human and mouse. Shown are G+C content in 50Kb segments of orthologous human and mouse genome segments. Spearman correlation is given on the plot. (C) Residual ToR is conserved. We computed the residual ToR (see Materials and Methods) for each of the experiments by subtracting the G+C to ToR trend (depicted in 2A) from the original ToR value. The residual profiles therefore lack any correlation to the regional G+C content. Shown are two-way comparisons among the residual profiles, demonstrating highly significant ToR correlation even after the G+C correlation has been normalized.

Replication time was previously shown to be correlated with the genomic regional G+C content [7], [12]–[14]. Indeed, we observe a strong correlation between ToR and G+C content in both human and mouse (Figure 2A, Figure S10). The regional G+C content is known to be conserved between mouse and human (Figure 2B). On the other hand, ToR structure is known to affect mutability and may therefore contribute to G+C content heterogeneity [32], [33]. To test if conservation of ToR and G+C content are two aspects of the same phenomenon we subtracted from the ToR of each probe the mean of ToR in probes with similar G+C contents, forming a *residual ToR* profile that was uncorrelated with the G+C content by design (see Materials and Methods, Figure 2C). We found that the residual ToR profiles are still highly correlated between mouse and human, which demonstrates that the conservation of ToR between species is not a mere consequence of slow G+C content divergence. Furthermore, the independence of ToR conservation from G+C conservation is supported by the conservation of tissue specific differences in ToR (discussed above), since such tissue specific differences cannot possibly be a direct consequence of G+C content. We did not find a significant correlation between ToR conservation and sequence conservation (see Materials and Methods), which suggests that ToR conservation is not reflecting global sequence conservation, but rather conservation of short subsequences at specific regulatory elements.

### ToR and chromosomal interactions

**Figure 3:**
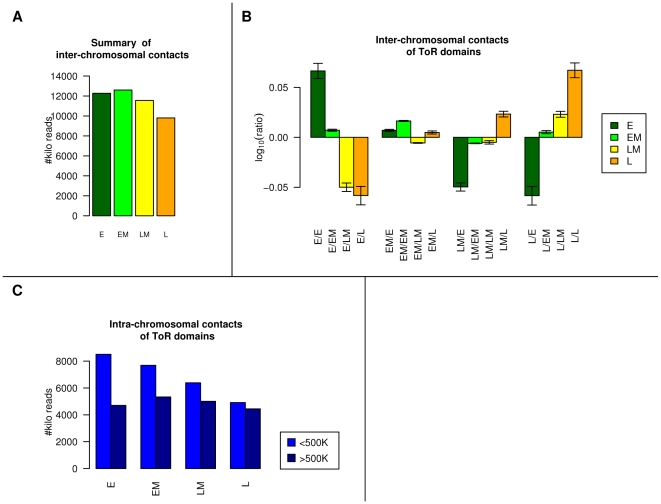
ToR and chromosomal interactions. (A) Late replicating domains are less accessible. The number of inter chromosomal interactions involving each replication time group are shown. We show the for each replication group the number of trans interactions that include this group (we call this measure the *accessibility* of the group). The late replicating group takes part in less interactions. (B) Regions with similar ToR tend to trans-interact. We show for each pair of replication groups the *interaction ratio* (log scale), which is the number of *trans* interactions, normalized according to the accessibility of the group (shown in Figure 3A). There is a bias towards self interactions within the early and the late replicating groups. (C) Early replicating DNA is more involved in close *cis* interaction. We show for each ToR group the number of intra-chromosomal interactions, divided into close interactions (<500K) and far interactions (>500K).

The conserved large-scale genomic ToR domains we have characterized, with their correlation to different genomic features, are likely to represent physical chromosomal domains with specific nuclear preferences. We analyzed published Hi-C chromosomal interaction data [15], which was measured on a human lymphoblastoid cell line (GM06990), and tested the interaction preferences of 4 equal-sized groups, each replicating in one of the quarters of the S phase. We measured the amount of interactions (paired-end reads) within groups and between groups, and studied it separately for intra- and inter-chromosomal interactions (Materials and Methods). We first found that late replicating domains are generally less represented in the Hi-C dataset, either due to their relative isolation or due to technical issues with chromatin extraction and shearing (Figure 3A). After normalizing this effect, we found that domains with similar ToR tend to *trans*-interact with each other more often than with domains with different ToRs (Figure 3B). When examining intra-chromosomal interactions, we found that early replicating domains have more Hi-C interactions than late replicating domains (Figure 3C). Interestingly, the additional chromosomal interactions of early replicating domains are primarily short-ranged (<500kb). This result is in agreement with the previously noted distributions of interaction distances for open (early replicating) and closed (late replicating) chromosomal domains [15]. The interactions of late replicating domains are relatively more biased toward long distances, while more of the interactions of early domains are representing local interactions.

### ToR and genome rearrangements

**Figure 4:**
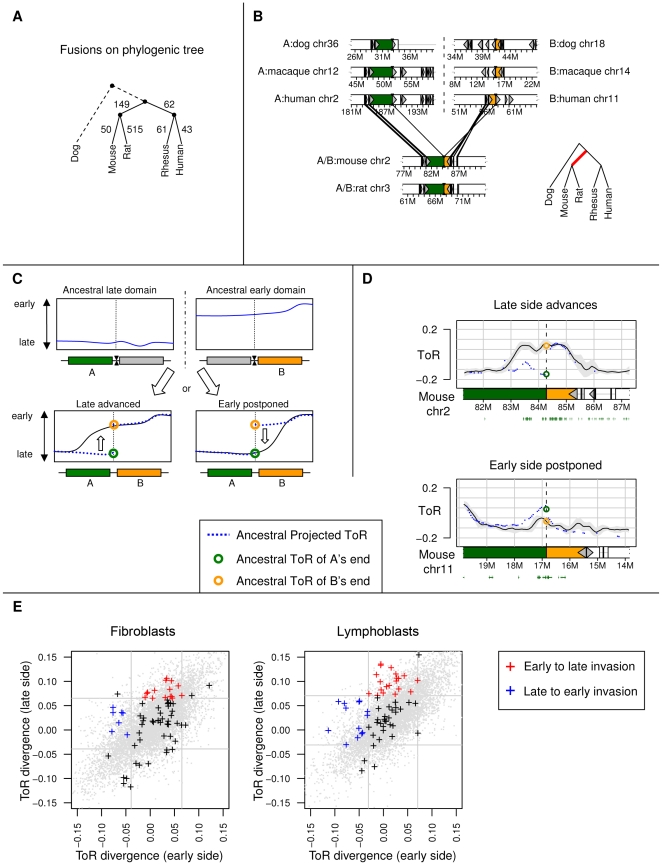
ToR divergence at genome rearrangement sites. (A) Phylogenetic tree. The phylogenetic tree used in our analysis, showing the number of simple fusion events on each branch. (B) Fusion event illustrated. Two syntenic blocks (colored green and orange) are adjacent in mouse and rat, and distal in human dog and rhesus. The branch associated with the event is marked in red on the phylo-tree. (C) Two possible divergence patterns following a fusion event. On top we show schematically the ancestral ToR of two distal segments (early replicating and late replicating domains) prior to fusion. After the fusion event the ToR can either propagate from the early domain into the late domain (early-to-late invasion), or from the late domain into the early domain (late-to-early invasion). (D) Invasion examples. We depict the mouse lymphoblast ToR with a black line (confidence intervals are shown in grey). The human ToR as projected onto the mouse genome is depicted with blue dots. The two segments that got fused are colored green (left segment) and orange (right segment). The approximated ToR near the breakpoint prior to fusion is depicted with a colored circle (green and orange) for both segments. Known genes are depicted with green rectangles below each graph. On top we show an example of the more common case of early-to-late invasion, while on bottom we show a late-to-early invasion (see Figure S12 for data on both cell types and more details). (E) ToR divergence at distal fusion sites. Shown is a scatter plot of the ToR divergence on the late side (segment that had later ToR prior to fusion, Y axis) versus the ToR divergence on the early side (segment that had earlier ToR prior to fusion, X axis). We draw the mean ToR divergence ± its standard deviation as vertical and horizontal grid lines (gray). We classify an event as an early-to-late invasion (E2L) if on the early side the divergence is close to the mean divergence (up to the standard deviation), and the late side divergence is greater than the sum of the mean divergence and its standard deviation (colored red). Similarly, we classify an event as an late-to-early (L2E) invasion if the late side the divergence is close to the mean divergence (up to the standard deviation), and the early side divergence is smaller than the mean divergence minus its standard deviation (colored blue). For fibroblasts we counted 15 E2L events versus 7 L2E events. For lymphoblasts we counted 23 E2L events versus 14 L2E events. In all cases we computed a hyper-geometric test versus 10,000 random points in the genome (plotted in gray) to verify that these counts are significantly diverged from the background (for L2E P value <10^−5^, for E2L P value <0.025).

Since the ToR domain structure of the human and mouse genome is highly conserved in alignable regions, we next focused on the conservation and divergence patterns near breakpoints. Rearrangements continuously reorganize the large-scale layout of the genome through translocations, inversions and duplications. Such events may shuttle a genomic region from one genomic context into an entirely different one. The dynamics of ToR divergence that follow rearrangements can hint at the mechanisms that regulate ToR. If replication initiation is mostly determined by local elements we can expect low ToR divergence even around rearrangements, but if the chromosomal neighborhood is a major factor in ToR regulation we would expect significant ToR divergence there. We used the inferCARs algorithm [34] to extract a collection of 1382 syntenic blocks (>50Kb) shared by human, rhesus, mouse, rat and dog, which cover over 92% of the human genome. Using dog as an outgroup, we identified 880 *simple fusion events* (Materials and Methods), which are events that can be associated to a unique branch in the phylogenetic tree. In Figure 4A we show the phylo-tree and the number of events on each branch. Most of these events are between domains of similar ToR (Figure S11), yet we are interested in events that fused domains of different ToR. An example of a simple event that is assigned to the common mouse-rat lineage branch is shown in Figure 4B. We considered two alternative scenarios for ToR divergence following fusion of an early replicating domain and a late replicating domain (Figure 4C). The first scenario involves an early-to-late invasion, where the late side accommodates and advances its replication. The opposite scenario involves late-to-early invasion, where the replication of the originally early domain is delayed following fusion. Examples of both types of invasions are given in Figure 4D. Analysis of all simple fusion events (Figure 4E) indicated that near breakpoints, ToR is more diverged than expected by chance (with more cases than expected representing significant divergence near fusion points, hyper geometric P<0.00015). This analysis provided us with a detailed list of genomic regions that went through ToR divergence following a change in genomic context (Table S1), opening new evolutionary avenues for further refining our understanding of ToR regulation. For example, we note that early-to-late invasion is more common than late-to-early invasion (15 versus 7 events for fibroblasts, 23 versus 14 events for lymphoblasts, see Figure S12 for more examples on both cell types). The mechanisms underlying ToR divergence can only be hypothesized given the current data (ToR measurements of a third outgroup species is needed to reconstruct evolutionary histories with higher certainty). For a subset of the early-to-late invasions (e.g., Figure S12C) the most simple mechanism, in which a single replication fork crosses the fusion point, is a valid explanation. In other cases, (e.g., Figure 4D), divergence encompass a territory that is much larger than the scope of a single fork. Importantly, despite these specific cases, genome rearrangements are not causing massive divergence of replication timing, and the overall replication structure is largely conserved between human and mouse, suggesting evolution typically shuffles ToR domains rather than breaking and fusing them.

### Distal rearrangements are preferentially fusing early replicating domains and are enriched for distal Hi-C interactions

**Figure 5:**
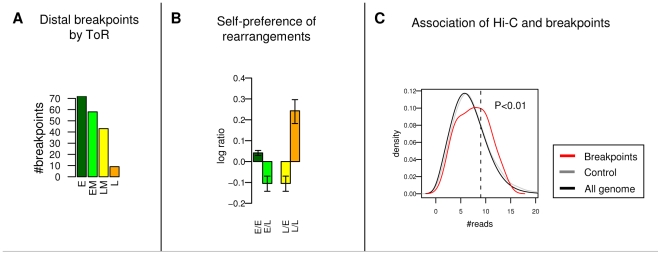
ToR and Hi-C preferences of distal rearrangements. (A) Distal fusion events involve early replicating domains. Each distal murine fusion event (inter-chromosomal or spanning at least 5MB in all non-rodent species) is associated with 2 human ToR groups (of the two segment ends that got fused). We show the breakdown of fusion events according to ToR groups. (B) Distal fusion events bring together domains with similar ToR. We split the genome to two equal groups (E:early, L:late), and counted for all murine distal fusions the number interaction of all possible pairs (E-E, E-L, L-L). We show the ratio between the counts and a random control (log_10_ scale), showing a significant preference for fusions of the same ToR group. (C) Distal fusion events are enriched for Hi-C interactions. We focused on murine distal fusion event that involves a pair of human sites that reside on different chromosomes. For each pair we counted the number of Hi-C interactions in the human genome (between segments of 1Mb centered on the breakpoint), which reflects chromosomal proximity. As a control we shuffled the pairs, getting random pairs of sites in the human genome that reside on different chromosomes. Using 75% as a threshold (dashed line), we tagged each pair as *interacting* (1st quartile) or *non-interacting* (other quartiles). Breakpoints are enriched in the interacting group (1.6 enrichment and P<0.01 in a Hyper geometric test). Shown are density plots of the number of reads between mouse fused pairs (red), between the shuffled control pairs (grey), and between a collection of random pairs selected over all of the genome (black).

We focused next on fusion events in the mouse lineage that involve segments that are distal (located more than 5Mb apart or on a different chromosome) in both human, dog and rhesus. We observed that these events preferentially involve early replicating domains (Figure 5A). Furthermore, rearrangements preferentially bring together genomic fragments of similar ToR (Figure 5B). This may be a consequence of a preference for rearrangements that involve early replicating regions, or a general mechanistic tendency to fuse breakpoints occurring at the same time in S phase. Another possibility is that the fusion of segments with very different ToR is more frequently deleterious since it violates the overall organization of the genome. We limited the analysis further, focusing on 55 fusion events on the mouse lineage for which the two human domains reside on different chromosomes (while their mouse orthologs are adjacent), and examined the level of interaction between the segments' ends, as reflected in the Hi-C dataset. We found that the interaction probability between these specific set of pairs is above the background (Figure 5C), which suggests that rearrangements occur between parts of the genome that are more often occupying the same nuclear compartment. Taken together, replication timing and Hi-C data suggest that genome rearrangements are correlated with the replication and nuclear architecture at an evolutionary scale, and that breakpoints generally shuffle genomic segments with similar ToR and prior chromosomal proximity. We anticipate that future data on chromosomal interactions at higher resolution and for additional species will allow a more quantitative estimation of the effect of replication timing and physical interactions on rearrangement rates.

## Discussion

We have shown that the mammalian genome is subject to a conserved replication timing structure that divides the genome into megabase-scale segments of coordinately replicating sequences. As suggested before [7], [8], this structure is highly correlated with other genomic traits (G+C content, gene density) but we show that its conservation is independent of these features. The human and mouse genomes differ significantly in their chromosomal landscape, which is a result of dramatic events like chromosomal fusion, switch to acrocentric layout and numerous large-scale rearrangements [29]. If ToR regulation was influenced by the location in the one-dimensional chromosomal space, such changes should have resulted in major ToR divergence between mouse and human, and in fragmentation of the ToR domain structure. As such divergence is not observed there must be some mechanism preventing it from occurring.

The genomic features that regulate ToR locally are currently uncharacterized. In theory, such elements could actively regulate the ToR of their surrounding genomic domains, and their conservation may be a consequence of strong selection working to conserve a functionally important ToR landscape. Under these assumptions, ToR would serve as a scaffold for the emergence of domains of active genes, thereby explaining the correlation between gene activity and early replication. Alternatively, local ToR regulation may be a consequence of gene activity rather than an enabler of it. According to this scenario, ToR conservation may be an indirect result of the conservation of gene activity and not a participant in driving it. However, the high conservation of ToR in gene deserts regions suggests that this latter possibility cannot fully account for the data. The more common early-to-late invasions of ToR that we observed (Figure 4E) support the notion of a simple *in cis* mechanism in which early replicating domains are positively regulated to retain their ToR regardless of their genomic context, while late replicating domains are passively regulated by lack of predisposition to early replication.

The conserved ToR landscape and the global map of chromosome interactions of the human genome reveal how genome rearrangements interact with the chromosomal architecture of the genome in four dimensions (the nucleus space and time of replication). We showed that rearrangements tend to bring together domains with similar ToR, and that pairs of loci that were fused in the mouse lineage, tend to *trans*-interact in the human genome. One explanation for these observations is that the mechanisms of breakpoint and repair increase the likelihood of rearrangements that involve loci with similar replication timing, or prior chromosomal proximity. Indeed, chromosomal proximity was recently suggested to underlie the cancer-prone translocation TMPRSS2-ERG [35]. An alternative explanation for the association between ToR, chromosomal interactions and breakpoints, may be a selective constraint on rearrangements that would significantly change the nuclear architecture by moving a domain to a foreign genomic context. A large-scale and deleterious change in ToR due to translocation was observed in human lymphocytes in cytogenetic resolution [36]. Data from additional species would allow for true phylogenetic reconstruction of the ToR history in different parts of the genome, and could help to resolve and refine the above hypotheses. We expect such data to provide unique insights into the regulation of DNA replication and to expand significantly our understanding of this key aspect of genome organization.

## Materials and Methods

### Cell culture

Mouse L1210 lymphocytic leukemia cells (ATCC CCL219) were grown in CO2-independent L-15 medium supplemented with 2 gr/L dextrose. Human Molt-4 acute lymphoblastic leukemia cells (ATCC CRL-1582) were grown in RPMI. Mouse Embryonic cells (MEF) were grown in DMEM supplemented with 0.2% beta-mercaptoethanol and 1% NeAA (Non-Essentials Amino-Acids). Primary foreskin fibroblasts transfected with the h-TERT gene (FFT) were grown in RPMI. All cells were supplemented with 10% fetal bovine serum, penicillin G and streptomycin sulfate.

### Fluorescence-activated cell sorting and DNA extraction

Cells were washed twice with PBS, fixed with ethanol, stained with 5 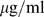 propidium iodide, and incubated with 50 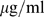 RNASE A. Then, the cells were sorted by their DNA content using the fluorescence-activated cell sorting (FACS) Vantage machine. DNA was extracted from S phase and G1 phase isolated cells using incubation with standard lysis buffer followed by Proteinase K treatment, phenol-chloroform extraction and ethanol precipitation. The resulting DNA was sonicated, cleaned using QIAGEN PCR purification kit and its concentration was measured using the Nano-Drop.

### DNA labeling and array hybridization

250ng-1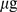 DNA isolated from G1 or S phase cells was labeled with dUTP-cy3 or dUTP-cy5 respectively, using Agilent's CGH labeling protocol (www.embl-heidelberg.de/courses/Agilent05/CGH-Protocol.pdf). Pairs of samples were co-hybridized on Agilent custom design mouse or human microarrays according to Agilent's hybridization protocol. The arrays were scanned using an Agilent scanner and raw data was analyzed using Agilent's feature extraction software. We have optimized the protocol to yield high quality data using as little as 250ng of input DNA (Figure S13). The ToR of each cell type was measured using two replicates (Figure S14).

### Microarray design

Arrays were designed using Agilent's website “eArray”. The mouse array covered the entire mouse genome with an average spacing of 40Kb and chromosome 19 with an average spacing of 1Kb (Agilent Microarray Design Identification (AMADID) #018925). The human array covered the entire human genome with an average spacing of 38Kb and all the sequenced part of chromosome 22 with an average spacing of 1Kb (AMADID #021214). All experiments were performed using Agilent's 2×105K CGH arrays. To allow simple comparison between human and mouse the data was binned to a 50Kb resolution, which resulted in ∼47,000 mouse bins and ∼53,000 human bins.

### Smoothing ToR data

To reduce noise on the single probe level (which is caused by dye bias [37]) we smoothed out the raw ToR data. We define the *smooth ToR* value of bin 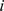 to be:
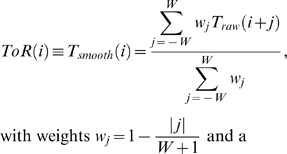
window size of 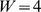. Throughout the paper we use the smooth ToR values. For the breakpoint analysis we define the (smooth) *right-sided ToR* to be:


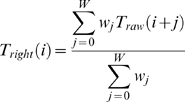
, with 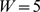 (*left-sided ToR* is defined in a similar fashion).

The confidence interval around the ToR profiles is the standard deviation of the 2W+1 values that were averaged when computing the smooth ToR profiles.

### Comparison of ToR and sequence conservation

For each 50Kb bin we defined the ToR conservation to be the difference between human fibroblast ToR and mouse fibroblast ToR. We defined the sequence conservation of the bin to be the percentage of conserved nucleotides between human and mouse (using *maf* files). Sequence conservation and ToR conservation are not correlated (Spearman 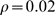).

### G+C analysis

We used the Human March 2006 Assembly (hg18) and the Mouse February 2006 Assembly (mm8) for human and mouse G+C content computation. To generate the G+C to ToR trend of Figure 2A we divided the G+C content spectrum (computed on windows with a width of 50Kb) between 0.2 to 0.7 into 50 equal sized segments [0.2, 0.21], [0.21, 0.22], …, [0.69,0.7], and computed the average ToR of each segment, which we call the *GC-predicted ToR* (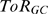). We defined the *residual ToR* to be: 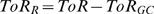. The residual ToR is “G+C normalized” in the sense that it has no significant correlation with the G+C content.

### Genomic mappings

We used the “liftOver” tool of UCSC [25]–[28] to project the ToR, the residual ToR and the regional G+C content of mouse onto the human genome. For each 50Kb mouse bin we defined a window of 20Kb centered on the middle of the bin, and attempted to project that window onto the human genome (with liftOver), requiring at least a 30% match, in order to obtain a high quality mapping. If a window succeeded projection we associated the original bin value to middle of the projected window (on human coordinates). In this manner we succeeded to project ∼30,000 bins (62%) onto the human genome, where we aligned them to the human 50Kb bins to allow easy comparison. We discarded all bins that had missing human data, leaving us with ∼27,000 bins. The result is a *comparative ToR map* that contains four aligned ToR profiles (FFT, MOLT4, MEF, L1210).

### Spatial clustering

The spatial clustering algorithm [31] works in an unsupervised fashion to identify contiguous genomic regions with similar trends of ToR in the two species and two tissues. We used as input for the algorithm the 4 ToR profiles of the ToR map. We normalized the profiles such that all had the same mean and standard deviation. We learned the most likely parameters of a star shaped hidden Markov model, with one central hidden non-emitting state, and N emitting states (the petals of the star), for N = 4,5,…,10. We noticed 4 states that appear in a robust manner for all N values, and therefore focused on the N = 4 model (data not shown).

### Comparison of ToR with various genomic features

We measured several genomic features for each of the ToR spatial clusters (Figure S9). Gene expression of FFT and Molt-4 were downloaded from UCSC [25]. Expression decreases with ToR, as previously shown. The Spearman correlation between the ToR difference and the gene expression difference (between FFT and Molt-4) is 0.11 (with a very small P value). ToR is correlated with distance to telomeres (Figure S9). We validated the known correlations between ToR and transcription density (amount of transcribed sequence, according to RefSeq genes), exon density, number of transcription start sites (in bins of 50Kb), amount of lamina interaction, and number of gene.

### Hi-C analysis

For the Hi-C analysis we represent ToR by the Molt-4 ToR track (Human lymphoblasts). We downloaded the Hi-C dataset [15] which is composed of ∼60M interacting pairs. We discarded interactions between loci without ToR data, which left us with ∼46M interactions; about half of them were inter-chromosomal and half were intra- chromosomal. We split the genome into 4 equal parts according to the FFT ToR quartiles (E:early, EM:early-medium, LM:late-medium, L:late). To generate Figure 3B, we counted for each possible pair of ToR groups the number of inter-chromosomal interactions. We then randomly paired all the loci that participated in an interaction to generate a background simulated interaction map, and computed for each pair a random count with its standard deviation. The ratio value for each pair is defined to be the number of counted interactions divided by the random count, and in the figure we display the log_10_ ratio. To generate Figure 3C we counted for each ToR group the number of intra-chromosomal close interactions (<500K) and far interactions (>500K). It should be noted that although the original Hi-C paper limits its analysis to 1Mb resolution, we were able to infer insights with higher resolution (<500Kb) since we were interested in four ToR categories, instead of in individual loci.

### Breakpoints

Using the algorithm of Ma et al. we identified 1382 homologous segments that are free of chromosome fissions or fusions as well as inversions or translocations larger than 50Kb. These long segments cover 92% of the human genome. In the phylogenetic tree of Figure 4A, we used only *simple events*, which are events that we could assign with high confidence to a unique branch of the phylotree (Figure S15, example in Figure 4B). Each event has a set of *posterior species* (leaves of the tree rooted below branch) and a set of *prior species* (leaves of tree rooted above branch). The *prior distance* of an event is the minimal distance between the two fused points among all *prior species*. In Table S1 we specify all events identified in this manner. We tagged all fusions with a prior distance greater than 5Mb as *distal events*, and tagged all other fusions as *close-range events*.

We focused on distal murine lineage events (events in the branches leading from the human-mouse ancestor to mouse). We computed ToR divergence on both sides of each fusion site. For each fusion, we defined the *late-side domain* to be the domain with the later human ToR (associated with 
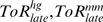
), and the *early-side domain* to be the domain with the earlier human ToR (associated with 
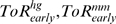
). We defined the *late divergence* and *early divergence* to be 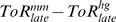 and 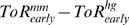, respectively. These two values reflect how much the mouse ToR has diverged from the ancestral ToR over evolution. For example, a positive late divergence (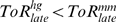) implies that after fusion the ToR of the murine domain advanced in time. In Figure 4E we show in a scatter plot late divergence versus early divergence. To generate Figure 5C we counted the number of Hi-C interactions in a window of 1MB between each pair distal simple fusion events in the mouse lineage. As control we shuffled all pairs, using only random pairs that reside on different chromosomes. Using the 75% percentile as a threshold, we tagged all pairs that were in the top quartile as *interacting* pairs and tagged all other pairs as *non-interacting* pairs. The original breakpoint pairs were enriched in the interacting group (1.6 enrichment hyper geometric P<0.01) when compared to the shuffled control pseudo breakpoints. We show the tagging threshold as a dashed line in Figure 5C.

### URL and accession numbers

ToR data was deposited in the GEO database, accession GSE17236.

## Supporting Information

Figure S1 Measuring ToR using DNA content of S and G1 phases. (A) A schematic representation of the changes in the copy numbers of early (E) and late (L) replicating regions along the cell cycle. Note that the DNA content of all regions in G1 is 1 whereas in S cells early regions DNA content can reach almost 2 and late regions DNA content is closer to 1. (B) In order to measure the ToR, G1 and S phase cells are isolated using a fluorescence activated cell sorter (FACS). DNA from those cells is labeled with fluorescence dyes and hybridized to custom design microarrays.
(1.12 MB TIF)

Figure S2 Comparison of the ToR generated by two different methods. (A) Smoothed (window size = 5) S/G1 log ratio data measured in L1210 cells (in the current paper) are shown along with the TR50 values (capturing the time (in minutes) of 50% cumulative replication) of the same regions measured previously by the isolation of newly replicated DNA at multiple time points along the S phase [7]. Note the agreement between the ToR from the two different data sources. (B) A comparison between all probes that have a ToR assignment in the current and published data shows a high Spearman correlation of ρ = −0.8 (insignificant P value).
(1.25 MB TIF)

Figure S3 Autocorrelation of ToR with varying window size. The x-axis is the number of probes we shift along the genome, and the y-axis is the autocorrelation between the probes and the shifted probes. The top 4 graphs are for the densely tiled human chromosome 22 and mouse chromosome 19, and the bottom 4 graphs are for all other chromosomes. To facilitate comparison we mark with dashed line the shift which equals 2Mb on all graphs. Note that since the densely sampled chromosomes have only a few domains the result correlation does not tend to zero, but instead retains a wavy form.
(0.31 MB TIF)

Figure S4 Conservation of time of replication in human and mouse cells. An expanded version of Figure 1A, showing ToR profiles for human fibroblasts and lymphoblasts and their corresponding projected mouse ToR profiles. Below each chromosome we show the human-mouse synteny map, color coded according to the corresponding mouse chromosomes.
(0.87 MB PDF)

Figure S5 Time of replication in mouse cells. Mouse ToR of the entire genome, displayed on mouse coordinates. Below each chromosome we show the human-mouse synteny map, color coded according to the corresponding human chromosomes.
(0.80 MB PDF)

Figure S6 ToR conservation as a function of exon density. We divided all bins into groups according to amount of exons they contain. We show the spearman correlation between human ToR and mouse ToR for each group. Below each bar we specify the amount of exons (between 0 and 1) and the percentage of the genome the group covers.
(0.26 MB TIF)

Figure S7 Large-scale domain structure of the replication landscape and sampling density. On the right we show ToR profiles (red) for the densely sampled chromosomes (1 probe per {similar, tilde operator }1Kb), with confidence intervals (grey). We then resampled the data, picking 1 probe out of each 50 probes, to get roughly one probe per 50Kb (like for the rest of the genome). We show on the left the sparse profiles thus computed (in blue), with confidence intervals (grey). Although placing more probes improves the profile quality, the large scale structure of the ToR profiles is clearly evident.
(0.42 MB TIF)

Figure S8 Diverged and conserved replication domains. Spatial clustering (see Materials and Methods) was used to dissect the genome in an unsupervised fashion into four clusters with common distributions across the four ToR profiles. Shown are the inferred clusters, representing early, medium, medium/late and late replication dynamics, which cover 92% of the data. The box plots on the left represent the clusters' ToR and G+C content distributions (light red for human, dark red for mouse). On the right is the full clustergram of the data, a visualization technique that display all clustered data in an orderly fashion. Each cluster refers to a different group of segments in the genome. We sort the segments according to length, place the longest segment on the bottom, and draw a separate box for each of the input tracks (Human/Mouse×Lymphoblasts/Fibroblasts). The width of the box is fixed to 3Mb and it is color-coded according to ToR (green - early replication, orange - late replication). Note that since the width is fixed to 3Mb we show for any segment that is shorter than 3Mb it's neighborhood (up to 3Mb).
(1.25 MB TIF)

Figure S9 Properties of spatial analysis clusters of Figure S8. For each of the 4 clusters we show gene expression (Kuhn, R.M., et al., The UCSC Genome Browser Database: update 2009), telomeric distance, transcription density (amount of transcribed sequence, according to RefSeq genes), exon density, number of transcription start sites (in bins of 50Kb), amount of lamina interaction [6]), and number of genes. 92% of the genome displayed distinct multivariate behavior. The other 8% were attributed to the background cluster, denoted by ‘B’ in the figure. Note that the background cluster is highly gene rich, reflecting the fact that ToR is less conserved in gene rich areas.
(0.25 MB TIF)

Figure S10 Negative correlation between G+C content and ToR. (A) The x-axis is the window width used to compute the G+C content, and the y-axis is the corresponding correlation between ToR and G+C content. Note that the correlation increases as the G+C window width increases up to windows of size 800Kb (marked with a dashed line), suggesting that ToR is better correlated with large scale G+C effects. (B) Correlation between ToR and G+C content (window = 50K) broken down by chromosomes.
(0.31 MB TIF)

Figure S11 Breakdown of fusion event. On the left we report the number of fusion events between different ToR groups. On the right we plot the number of fusions between similar ToR (i.e. L/L, LM/LM/EM/EM or E/E), the number of fusions between different ToR, and the number of events without ToR data. Note that between 48%–60% of the events occur between similar ToR. This is mainly due to the fact that most fusion events are close-ranged and ToR changes slowly along the genome.
(0.19 MB TIF)

Figure S12 Fusion examples. ToR divergence near near murine fusion sites. Gene density is marked with green, under each plot. We depict ToR with a black line (the confidence interval is shown in grey), and depict projected ToR with blue dots. The two segments that got fused in the mouse lineage are colored green (left segment) and orange (right segment). The approximated ToR near the breakpoint prior to fusion is depicted with a colored circle (green and orange) for both segments. (A,B) are detailed versions of Figure 4D. In (C,D) we show for both celltypes the top diverged events of both early-to-late invasion (red in Figure 4E) and late-to-early invasion (blue in Figure 4E).
(1.25 MB PDF)

Figure S13 Reproducibility of the DNA content method for ToR determination. ToR of mouse L1210 cells was determined in duplicate using either 250ng (purple dots) or 1000ng (blue dots) input DNA. The raw log ratios of probes along 90Mb of chromosome 1 probed with a 50Kb density (A) and of 20Mb of chromosome 19 probed with a 1Kb density (B) are shown. Note the high similarity between the duplicates. (C) A comparison between all probes that have a ToR assignment in the two experiments shows a high (ρ = 0.9) Spearman correlation (insignificant P value).
(1.94 MB TIF)

Figure S14 Experiment replicates. The ToR profile of each cell type was measured twice. All replicates were biological (completely separate experiment), except for the Mouse lymphoblasts for which both replicates were sorted in the same time. We show the spearman correlation on the figure.
(0.20 MB TIF)

Figure S15 Using an outgroup to identify certified rearrangement events. We show 4 syntenic block (colored A:green, B:orange, C:light grey, D:dark grey). We connect with dashed lines the homologuos intstances of the same block in different species. On top we demonstrate a simple fusion event. In species S1,S2 and Out (which serves as an outgroup) block A is adjacent to block C and block B is adjacent to block D. In species S3,S4 we have block A adjacent to block B (while blocks C,D got translocated to another place and are not shown). We therefore assign with high confidence a fusion event of A,B to the red branch in the phylotree. On the bottom we show a simple separation event. In that case the outgroup agrees with the species S3,S4 and we therefore regard the event as a separation event of A,B, and again we assign it to the red edge.
(0.18 MB TIF)

Table S1 List of breakpoints. List of 880 fusion events used in the analysis. For each fusion we show the associated branch in the phylo-tree, the human and the mouse coordinates, and the strand (direction in each genome where the syntenic block lies).
(0.19 MB XLS)
