# Supplementary figures and images for: Comparative Analysis of DNA Replication Timing Reveals Conserved Large-Scale Chromosomal Architecture
Source: PLoS Genet. 2010 Jul 1;6(7):e1001011. doi: 10.1371/journal.pgen.1001011 (PMC2895651; doi:10.1371/journal.pgen.1001011)

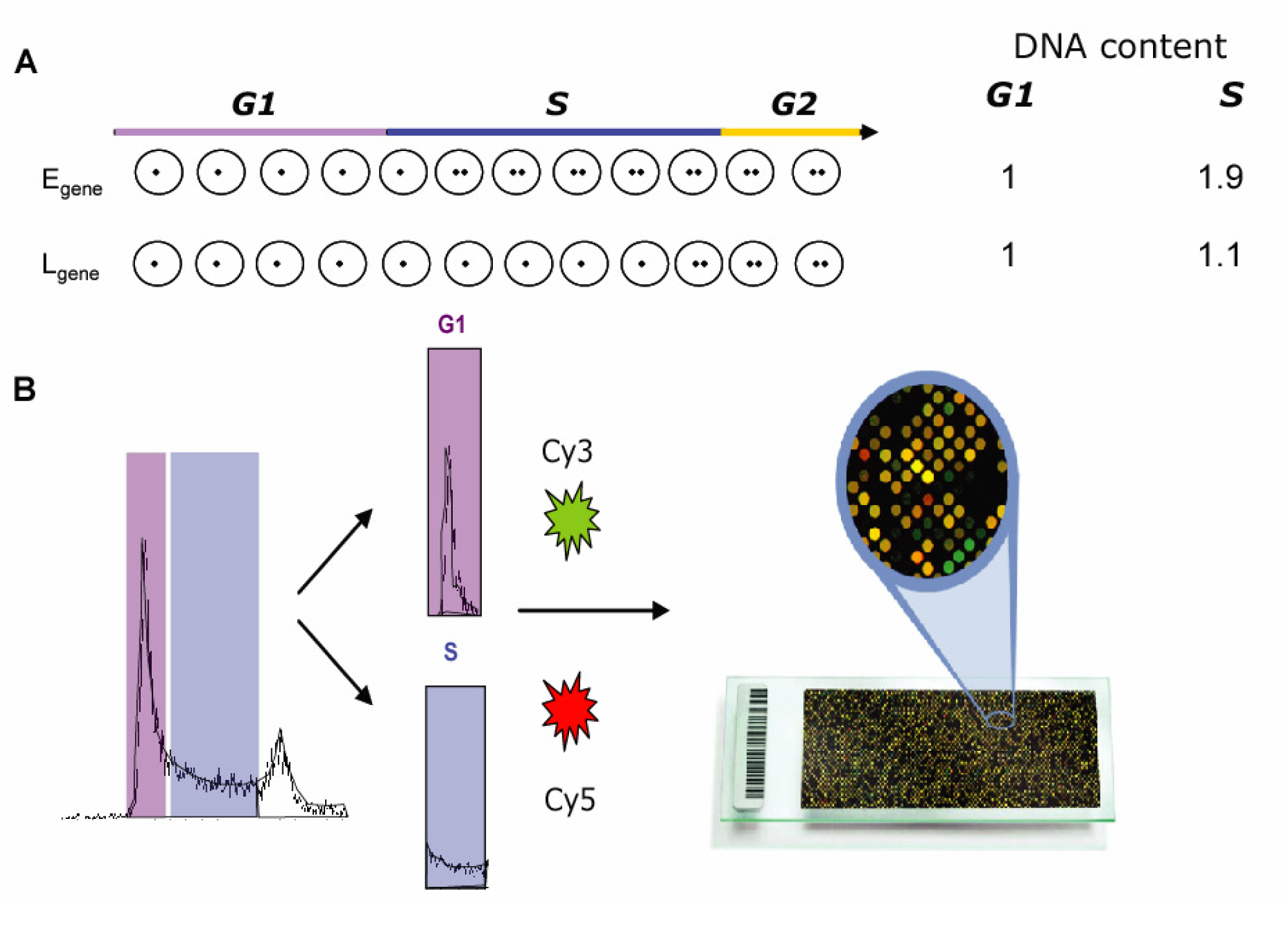

Supplement: Figure S1 — Measuring ToR using DNA content of S and G1 phases. (A) A schematic representation of the changes in the copy numbers of early (E) and late (L) replicating regions along the cell cycle. Note that the DNA content of all regions in G1 is 1 whereas in S cells early regions DNA content can reach almost 2 and late regions DNA content is closer to 1. (B) In order to measure the ToR, G1 and S phase cells are isolated using a fluorescence activated cell sorter (FACS). DNA from those cells is labeled with fluorescence dyes and hybridized to custom design microarrays. (1.12 MB TIF) [file pgen.1001011.s001.tif]

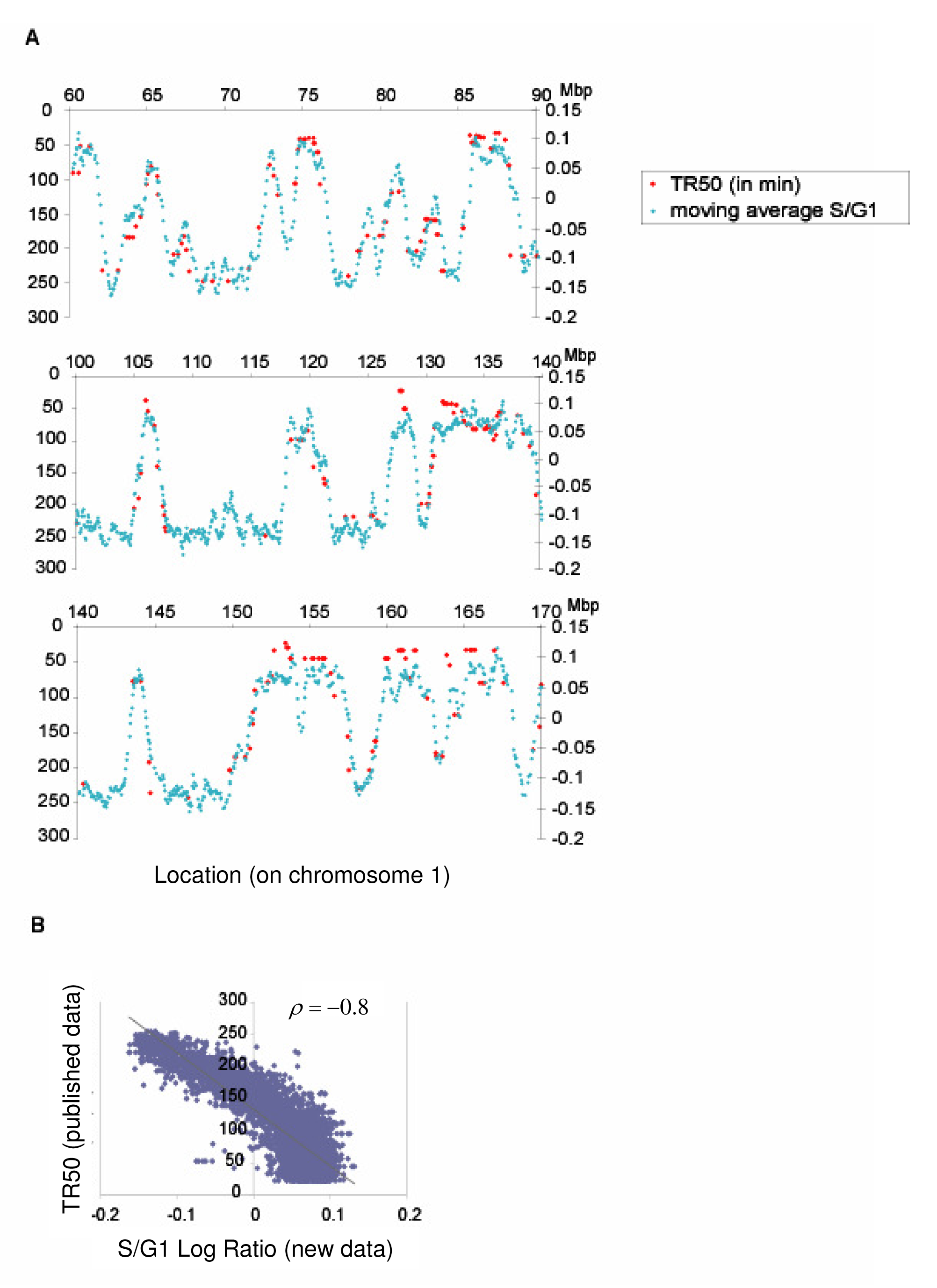

Supplement: Figure S2 — Comparison of the ToR generated by two different methods. (A) Smoothed (window size = 5) S/G1 log ratio data measured in L1210 cells (in the current paper) are shown along with the TR50 values (capturing the time (in minutes) of 50% cumulative replication) of the same regions measured previously by the isolation of newly replicated DNA at multiple time points along the S phase [7]. Note the agreement between the ToR from the two different data sources. (B) A comparison between all probes that have a ToR assignment in the current and published data shows a high Spearman correlation of ρ = −0.8 (insignificant P value). (1.25 MB TIF) [file pgen.1001011.s002.tif]

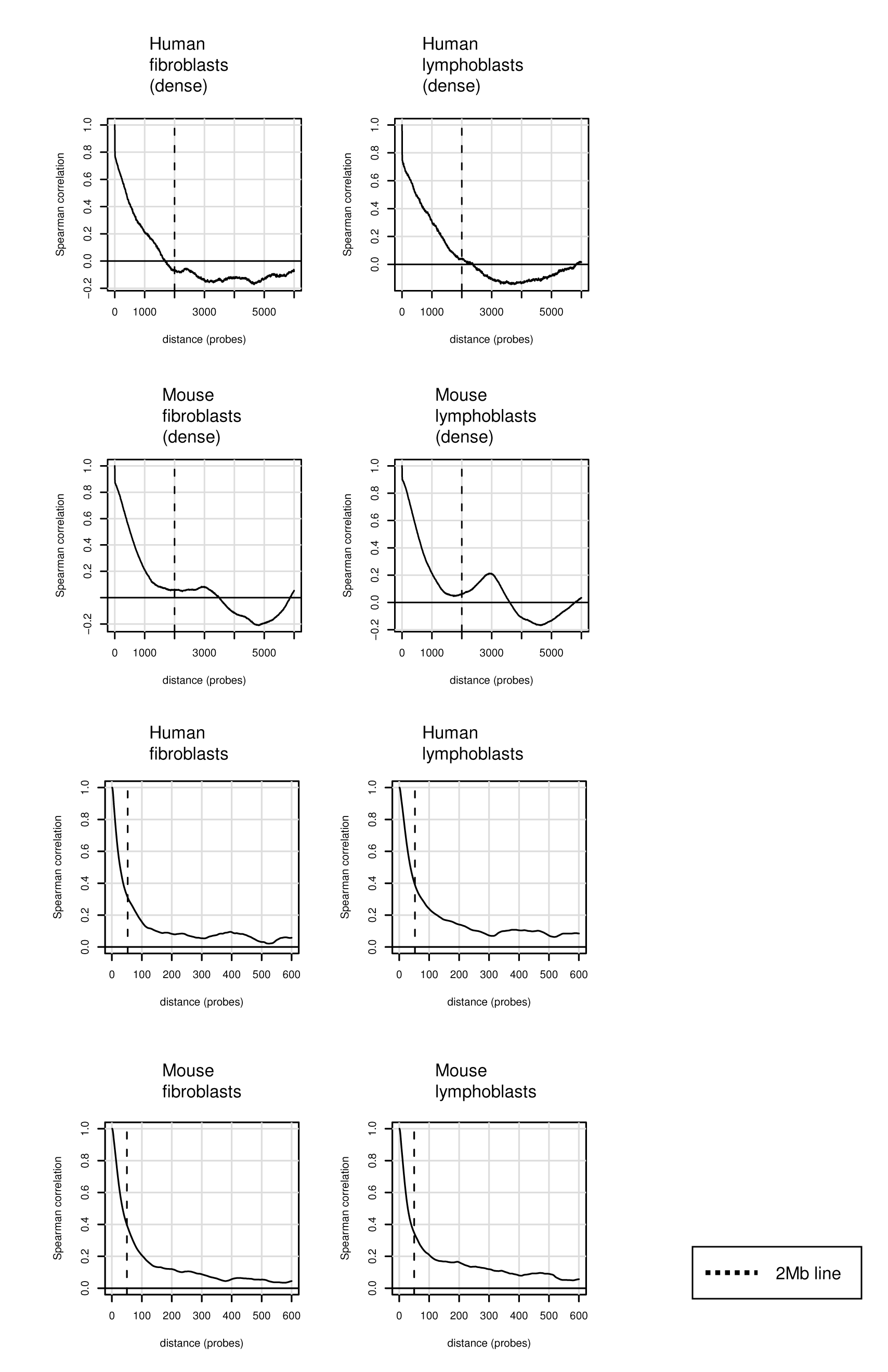

Supplement: Figure S3 — Autocorrelation of ToR with varying window size. The x-axis is the number of probes we shift along the genome, and the y-axis is the autocorrelation between the probes and the shifted probes. The top 4 graphs are for the densely tiled human chromosome 22 and mouse chromosome 19, and the bottom 4 graphs are for all other chromosomes. To facilitate comparison we mark with dashed line the shift which equals 2Mb on all graphs. Note that since the densely sampled chromosomes have only a few domains the result correlation does not tend to zero, but instead retains a wavy form. (0.31 MB TIF) [file pgen.1001011.s003.tif]

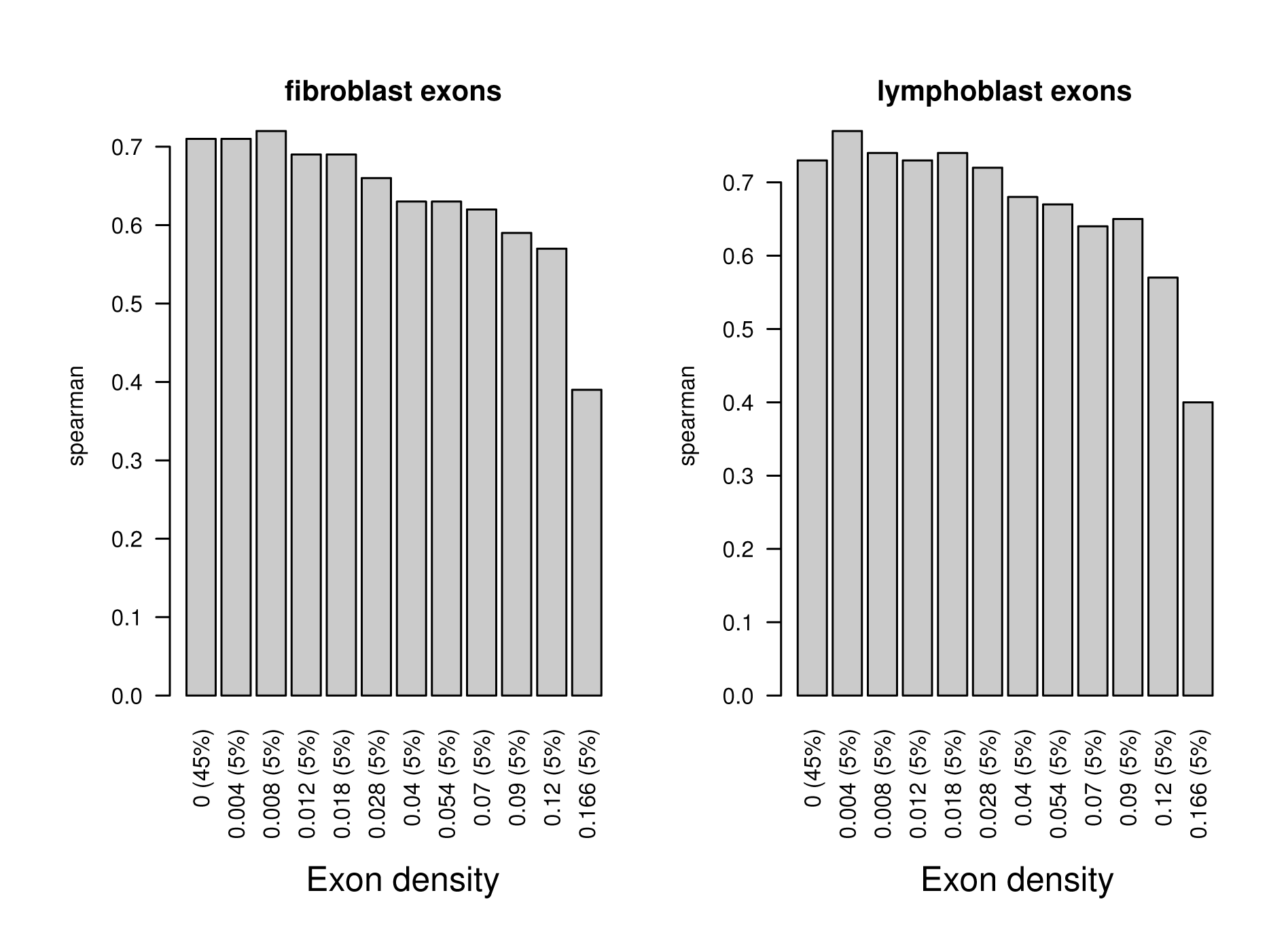

Supplement: Figure S6 — ToR conservation as a function of exon density. We divided all bins into groups according to amount of exons they contain. We show the spearman correlation between human ToR and mouse ToR for each group. Below each bar we specify the amount of exons (between 0 and 1) and the percentage of the genome the group covers. (0.26 MB TIF) [file pgen.1001011.s006.tif]

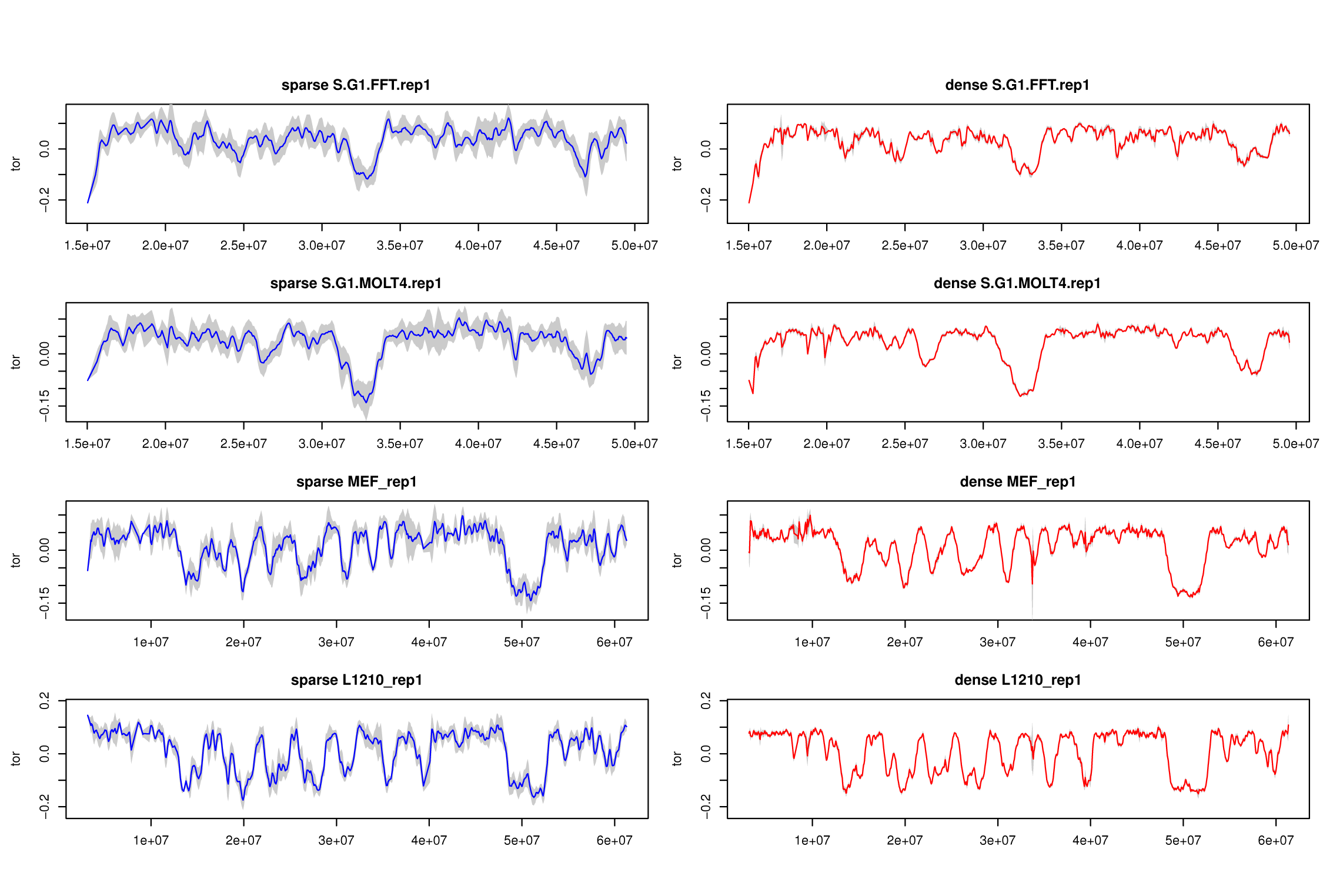

Supplement: Figure S7 — Large-scale domain structure of the replication landscape and sampling density. On the right we show ToR profiles (red) for the densely sampled chromosomes (1 probe per {similar, tilde operator }1Kb), with confidence intervals (grey). We then resampled the data, picking 1 probe out of each 50 probes, to get roughly one probe per 50Kb (like for the rest of the genome). We show on the left the sparse profiles thus computed (in blue), with confidence intervals (grey). Although placing more probes improves the profile quality, the large scale structure of the ToR profiles is clearly evident. (0.42 MB TIF) [file pgen.1001011.s007.tif]

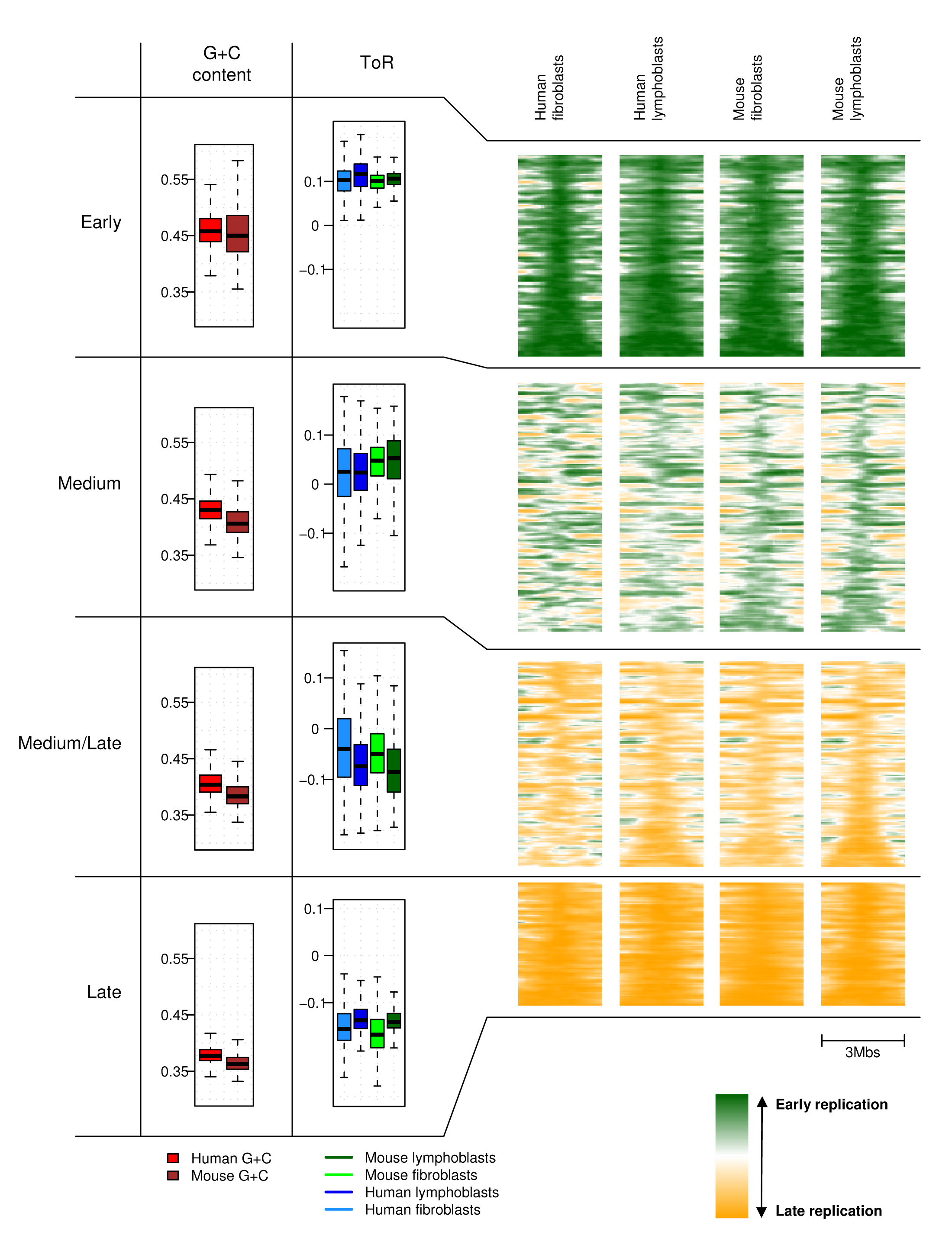

Supplement: Figure S8 — Diverged and conserved replication domains. Spatial clustering (see Materials and Methods) was used to dissect the genome in an unsupervised fashion into four clusters with common distributions across the four ToR profiles. Shown are the inferred clusters, representing early, medium, medium/late and late replication dynamics, which cover 92% of the data. The box plots on the left represent the clusters' ToR and G+C content distributions (light red for human, dark red for mouse). On the right is the full clustergram of the data, a visualization technique that display all clustered data in an orderly fashion. Each cluster refers to a different group of segments in the genome. We sort the segments according to length, place the longest segment on the bottom, and draw a separate box for each of the input tracks (Human/Mouse×Lymphoblasts/Fibroblasts). The width of the box is fixed to 3Mb and it is color-coded according to ToR (green - early replication, orange - late replication). Note that since the width is fixed to 3Mb we show for any segment that is shorter than 3Mb it's neighborhood (up to 3Mb). (1.25 MB TIF) [file pgen.1001011.s008.tif]

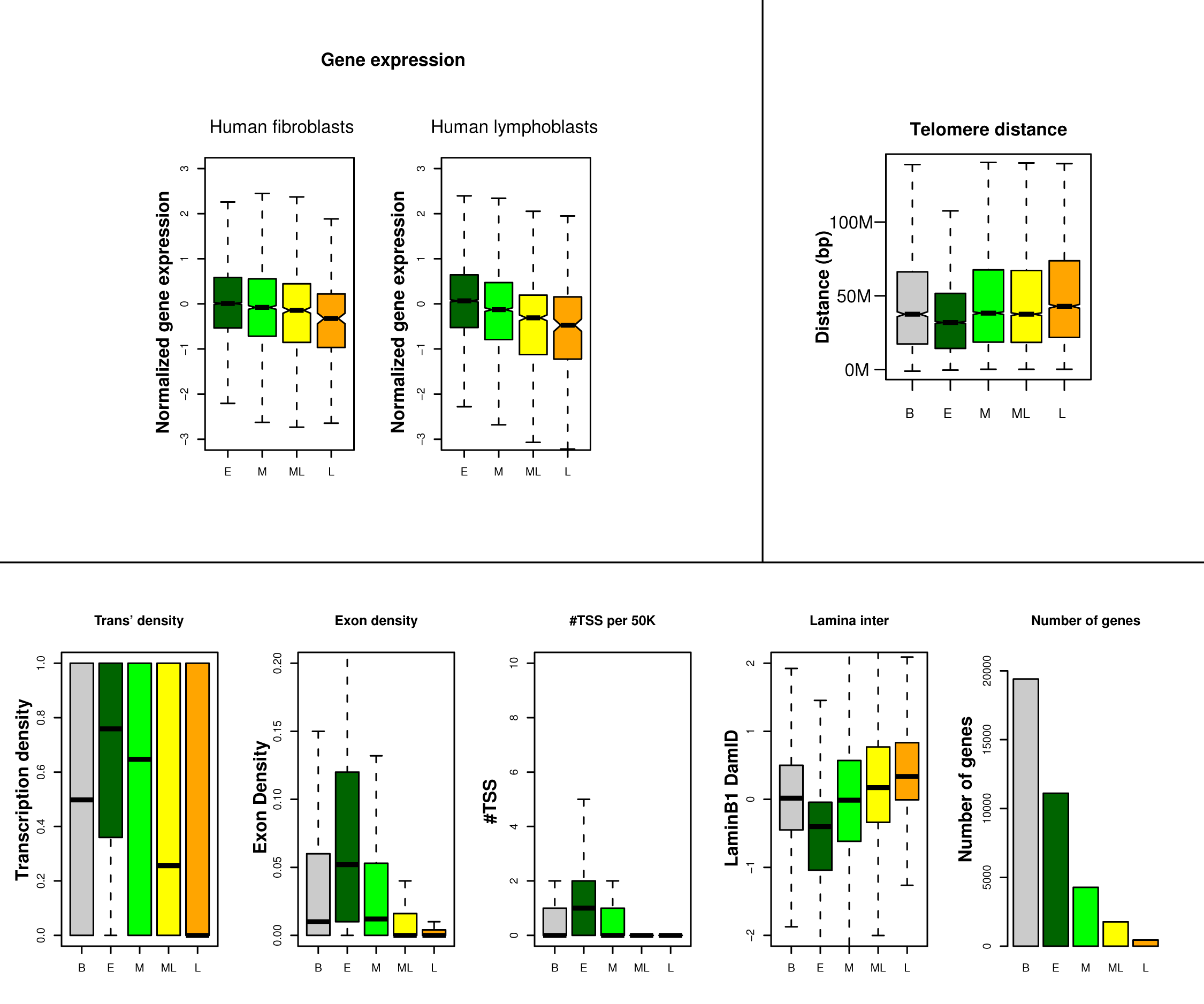

Supplement: Figure S9 — Properties of spatial analysis clusters of Figure S8. For each of the 4 clusters we show gene expression (Kuhn, R.M., et al., The UCSC Genome Browser Database: update 2009), telomeric distance, transcription density (amount of transcribed sequence, according to RefSeq genes), exon density, number of transcription start sites (in bins of 50Kb), amount of lamina interaction [6]), and number of genes. 92% of the genome displayed distinct multivariate behavior. The other 8% were attributed to the background cluster, denoted by ‘B’ in the figure. Note that the background cluster is highly gene rich, reflecting the fact that ToR is less conserved in gene rich areas. (0.25 MB TIF) [file pgen.1001011.s009.tif]

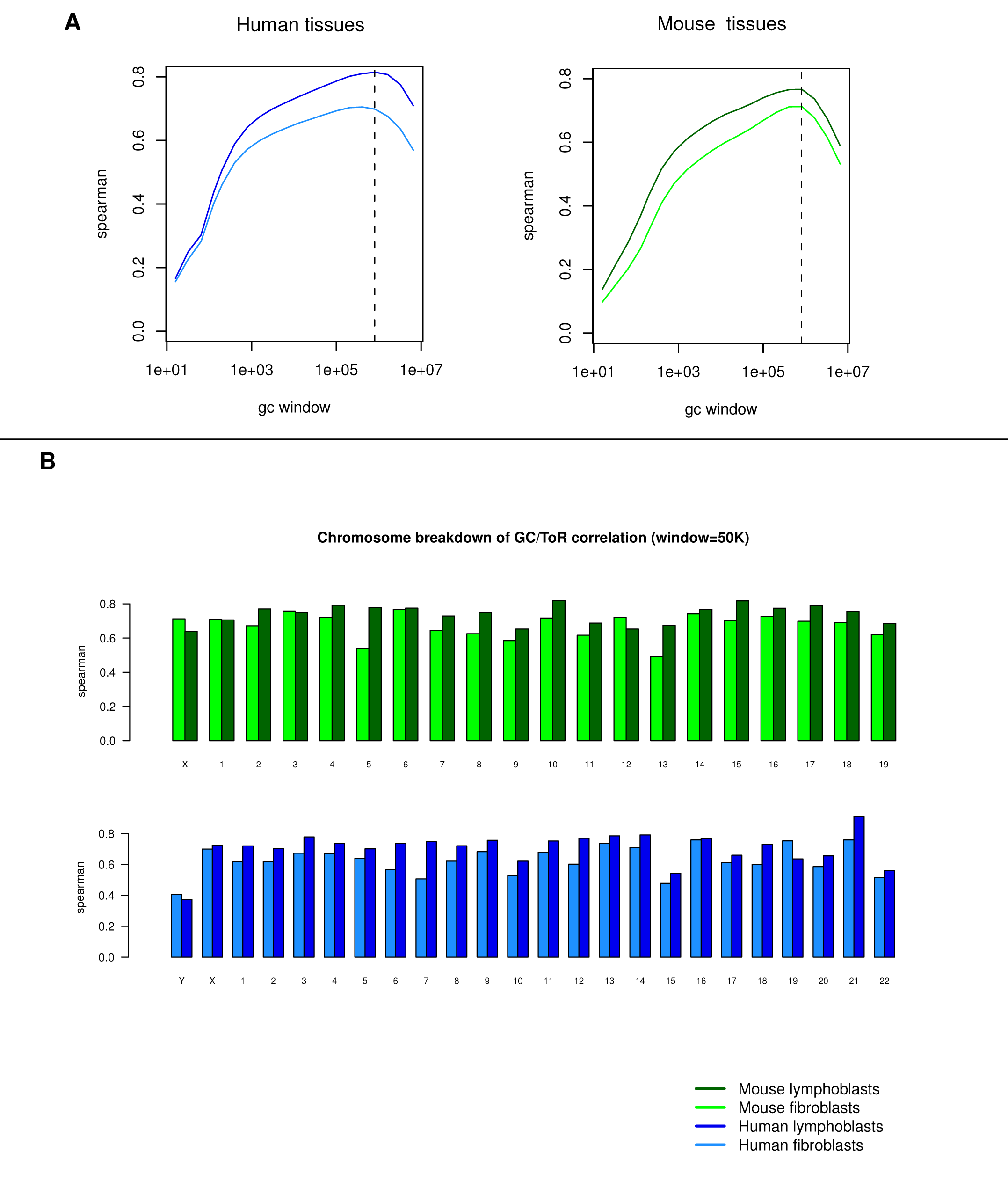

Supplement: Figure S10 — Negative correlation between G+C content and ToR. (A) The x-axis is the window width used to compute the G+C content, and the y-axis is the corresponding correlation between ToR and G+C content. Note that the correlation increases as the G+C window width increases up to windows of size 800Kb (marked with a dashed line), suggesting that ToR is better correlated with large scale G+C effects. (B) Correlation between ToR and G+C content (window = 50K) broken down by chromosomes. (0.31 MB TIF) [file pgen.1001011.s010.tif]

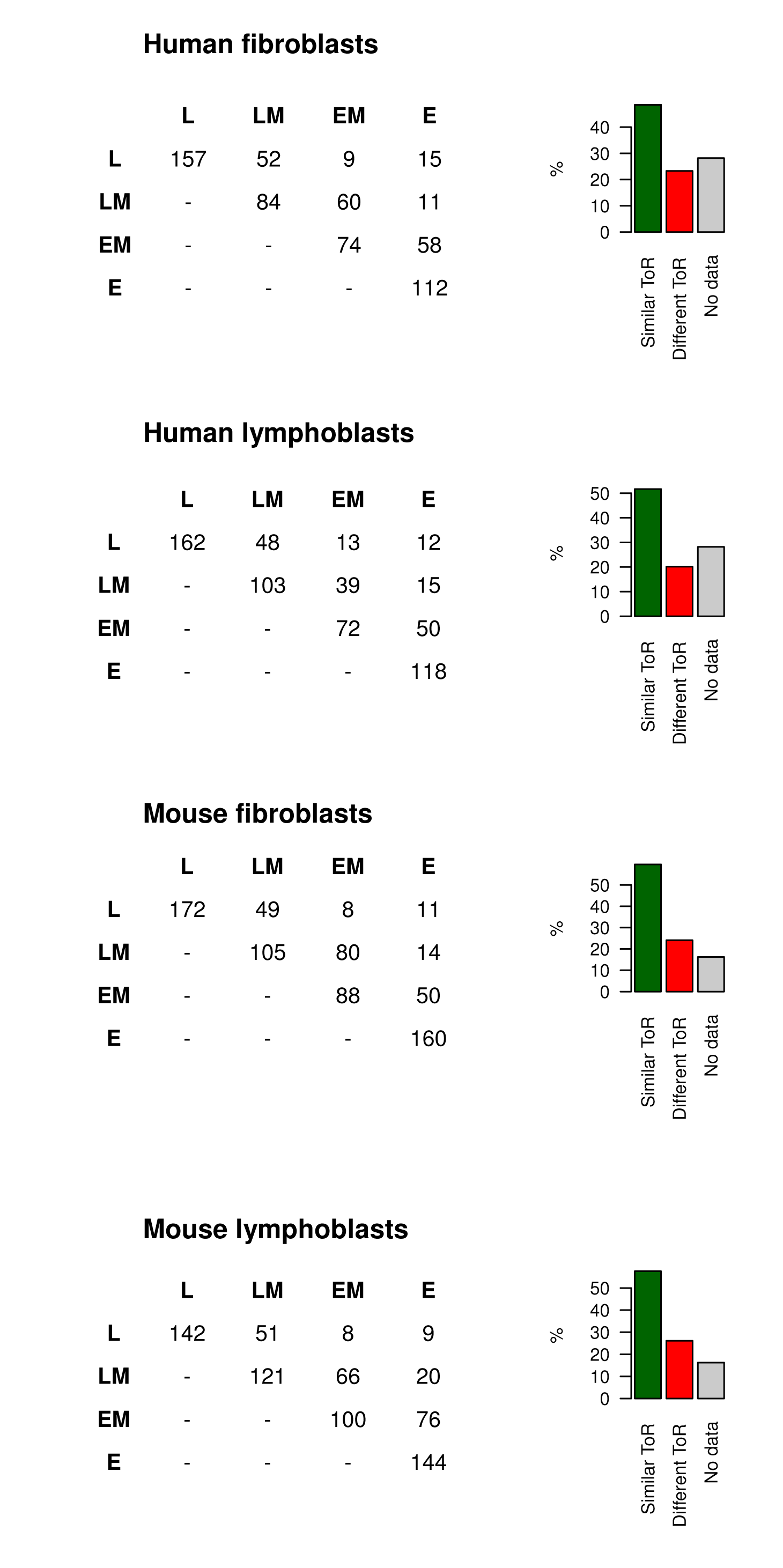

Supplement: Figure S11 — Breakdown of fusion event. On the left we report the number of fusion events between different ToR groups. On the right we plot the number of fusions between similar ToR (i.e. L/L, LM/LM/EM/EM or E/E), the number of fusions between different ToR, and the number of events without ToR data. Note that between 48%–60% of the events occur between similar ToR. This is mainly due to the fact that most fusion events are close-ranged and ToR changes slowly along the genome. (0.19 MB TIF) [file pgen.1001011.s011.tif]

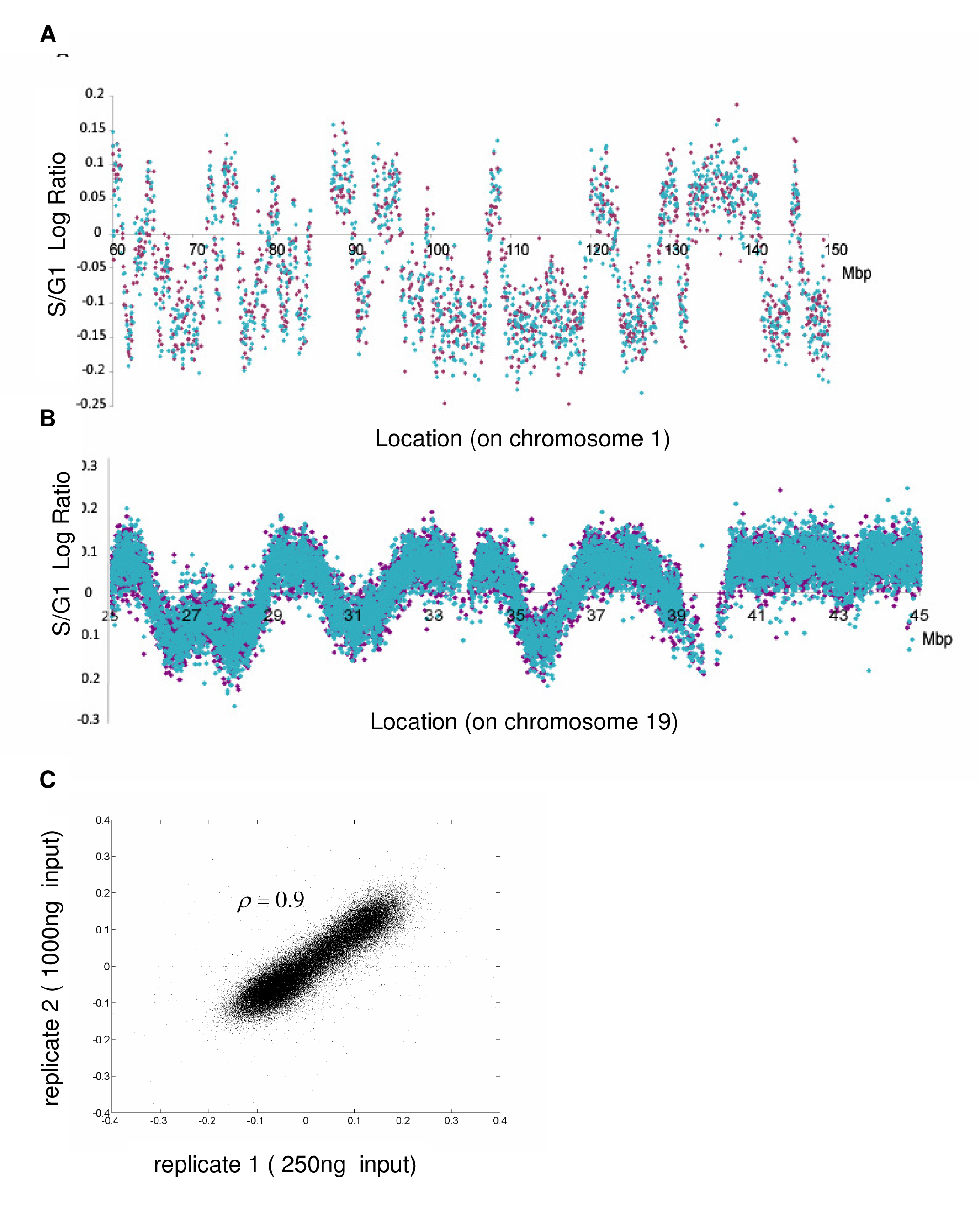

Supplement: Figure S13 — Reproducibility of the DNA content method for ToR determination. ToR of mouse L1210 cells was determined in duplicate using either 250ng (purple dots) or 1000ng (blue dots) input DNA. The raw log ratios of probes along 90Mb of chromosome 1 probed with a 50Kb density (A) and of 20Mb of chromosome 19 probed with a 1Kb density (B) are shown. Note the high similarity between the duplicates. (C) A comparison between all probes that have a ToR assignment in the two experiments shows a high (ρ = 0.9) Spearman correlation (insignificant P value). (1.94 MB TIF) [file pgen.1001011.s013.tif]

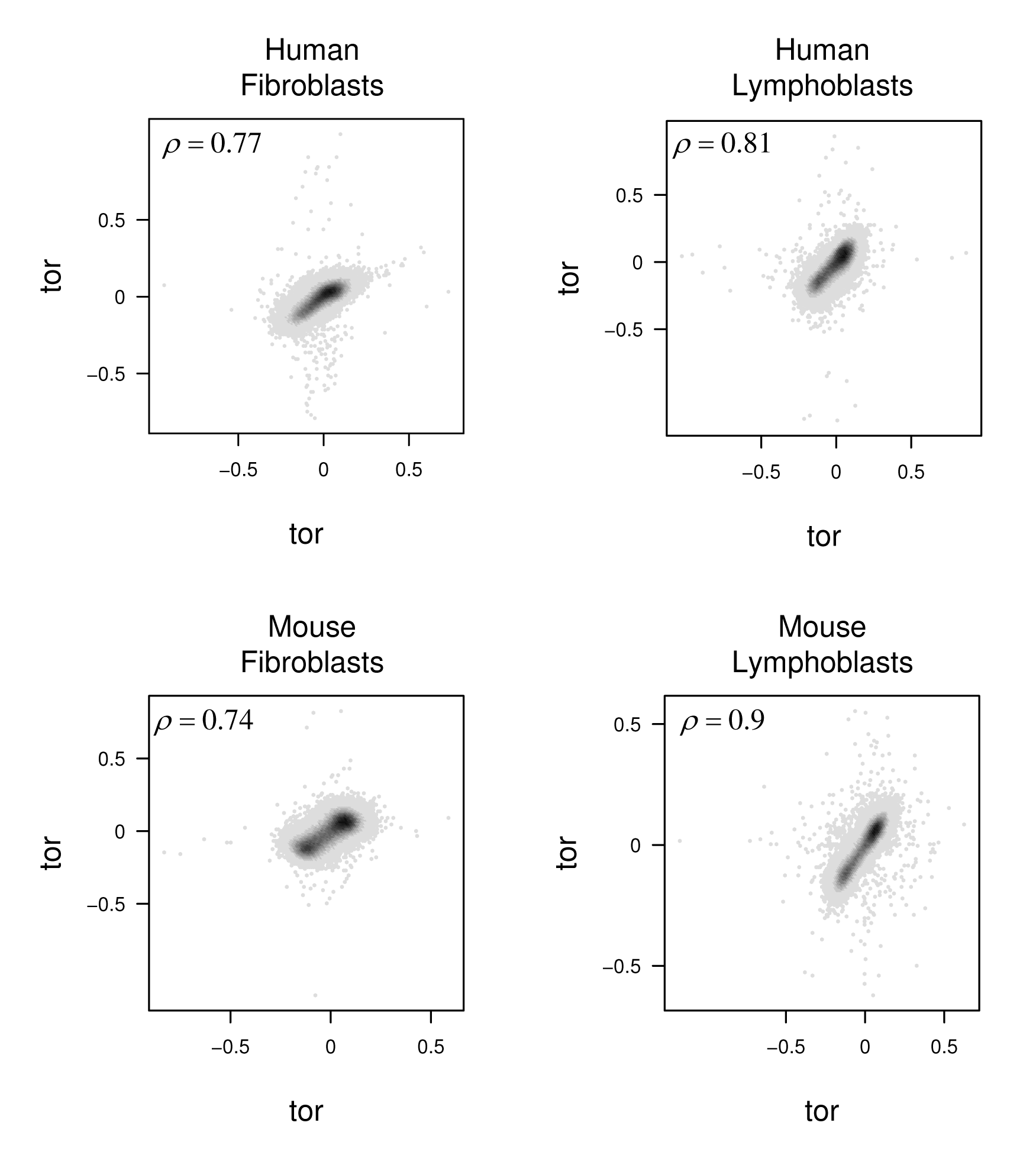

Supplement: Figure S14 — Experiment replicates. The ToR profile of each cell type was measured twice. All replicates were biological (completely separate experiment), except for the Mouse lymphoblasts for which both replicates were sorted in the same time. We show the spearman correlation on the figure. (0.20 MB TIF) [file pgen.1001011.s014.tif]

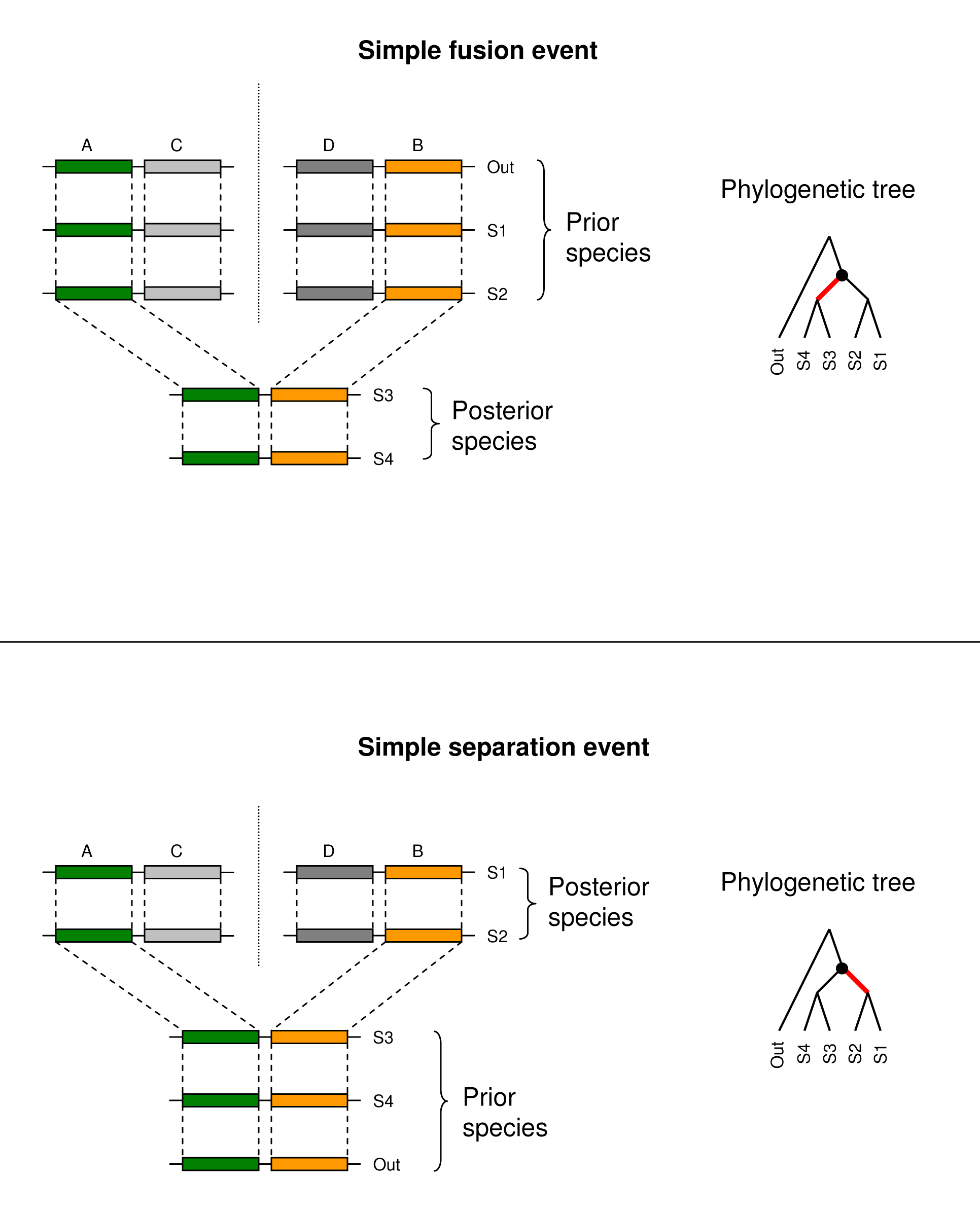

Supplement: Figure S15 — Using an outgroup to identify certified rearrangement events. We show 4 syntenic block (colored A:green, B:orange, C:light grey, D:dark grey). We connect with dashed lines the homologuos intstances of the same block in different species. On top we demonstrate a simple fusion event. In species S1,S2 and Out (which serves as an outgroup) block A is adjacent to block C and block B is adjacent to block D. In species S3,S4 we have block A adjacent to block B (while blocks C,D got translocated to another place and are not shown). We therefore assign with high confidence a fusion event of A,B to the red branch in the phylotree. On the bottom we show a simple separation event. In that case the outgroup agrees with the species S3,S4 and we therefore regard the event as a separation event of A,B, and again we assign it to the red edge. (0.18 MB TIF) [file pgen.1001011.s015.tif]
