# Supplementary material for: Comparative Analysis of DNA Replication Timing Reveals Conserved Large-Scale Chromosomal Architecture
Source: PLoS Genet. 2010 Jul 1;6(7):e1001011. doi: 10.1371/journal.pgen.1001011 (PMC2895651; doi:10.1371/journal.pgen.1001011)
Supplement: Figure S4 — Conservation of time of replication in human and mouse cells. An expanded version of Figure 1A, showing ToR profiles for human fibroblasts and lymphoblasts and their corresponding projected mouse ToR profiles. Below each chromosome we show the human-mouse synteny map, color coded according to the corresponding mouse chromosomes. (0.87 MB PDF) [file pgen.1001011.s004.pdf]

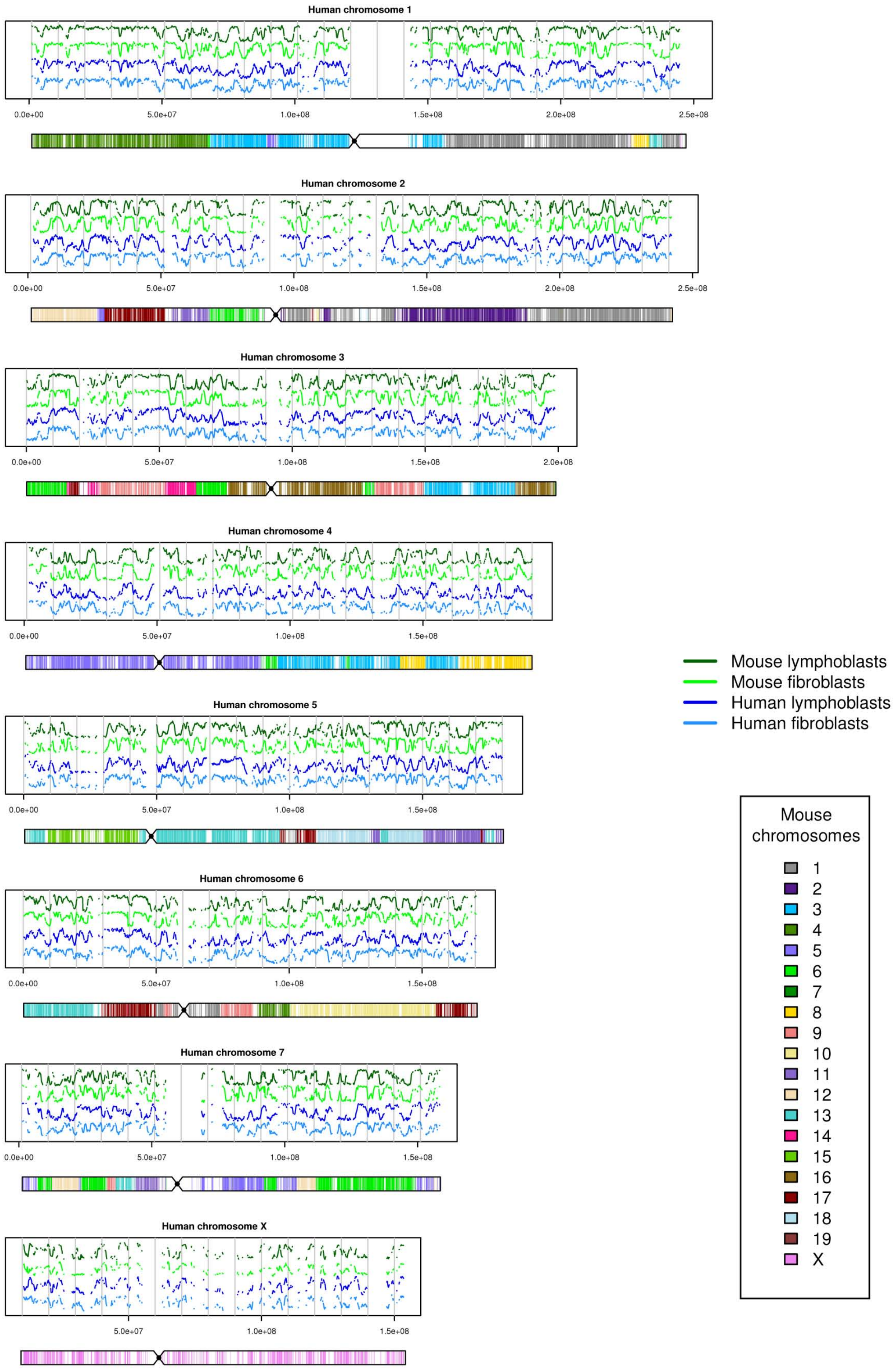

Human chromosome 1
0.0e+00
5.0e+07
1.0e+08
1.5e+08
2.0e+08
2.5e+08
Human chromosome 2
Human chromosome 3
Human chromosome 4
Human chromosome 5
Human chromosome 6
Human chromosome 7
Human chromosome X
Mouse lymphoblasts
Mouse fibroblasts
Human lymphoblasts
Human fibroblasts
Mouse chromosomes
1
2
3
4
5
6
7
8
9
10
11
12
13
14
15
16
17
18
19
X

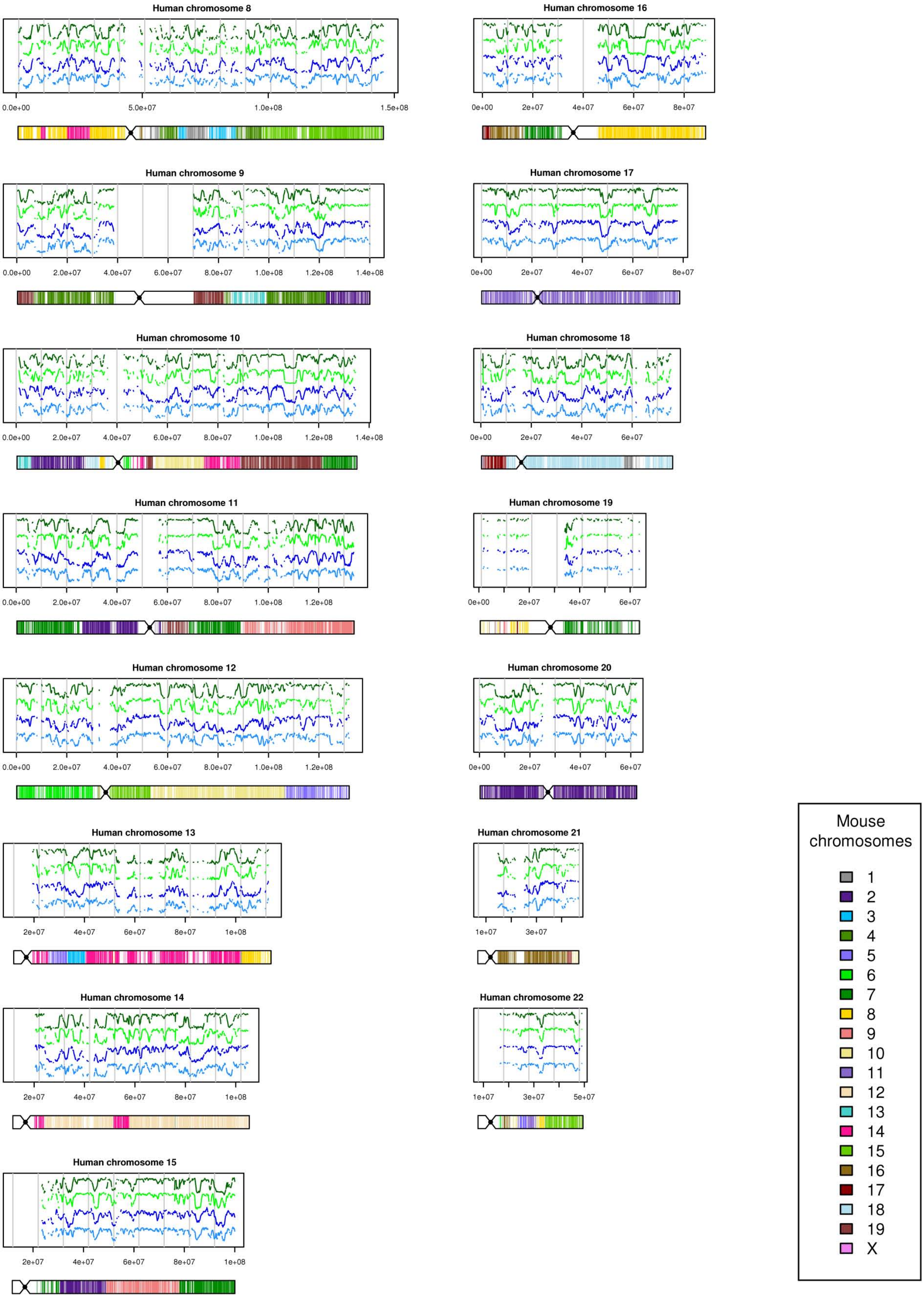
Human chromosome 8
0.0e+00 5.0e+07 1.0e+08 1.5e+08
Human chromosome 9
0.0e+00 2.0e+07 4.0e+07 6.0e+07 8.0e+07 1.0e+08 1.2e+08 1.4e+08
Human chromosome 10
0.0e+00 2.0e+07 4.0e+07 6.0e+07 8.0e+07 1.0e+08 1.2e+08 1.4e+08
Human chromosome 11
0.0e+00 2.0e+07 4.0e+07 6.0e+07 8.0e+07 1.0e+08 1.2e+08
Human chromosome 12
0.0e+00 2.0e+07 4.0e+07 6.0e+07 8.0e+07 1.0e+08 1.2e+08
Human chromosome 13
2e+07 4e+07 6e+07 8e+07 1e+08
Human chromosome 14
2e+07 4e+07 6e+07 8e+07 1e+08
Human chromosome 15
2e+07 4e+07 6e+07 8e+07 1e+08
Human chromosome 16
0e+00 2e+07 4e+07 6e+07 8e+07
Human chromosome 17
0e+00 2e+07 4e+07 6e+07 8e+07
Human chromosome 18
0e+00 2e+07 4e+07 6e+07
Human chromosome 19
0e+00 2e+07 4e+07 6e+07
Human chromosome 20
0e+00 2e+07 4e+07 6e+07
Human chromosome 21
1e+07 3e+07
Human chromosome 22
1e+07 3e+07 5e+07
Mouse chromosomes
1
2
3
4
5
6
7
8
9
10
11
12
13
14
15
16
17
18
19
X
