# Supplementary material for: Comparative Analysis of DNA Replication Timing Reveals Conserved Large-Scale Chromosomal Architecture
Source: PLoS Genet. 2010 Jul 1;6(7):e1001011. doi: 10.1371/journal.pgen.1001011 (PMC2895651; doi:10.1371/journal.pgen.1001011)
Supplement: Figure S5 — Time of replication in mouse cells. Mouse ToR of the entire genome, displayed on mouse coordinates. Below each chromosome we show the human-mouse synteny map, color coded according to the corresponding human chromosomes. (0.80 MB PDF) [file pgen.1001011.s005.pdf]

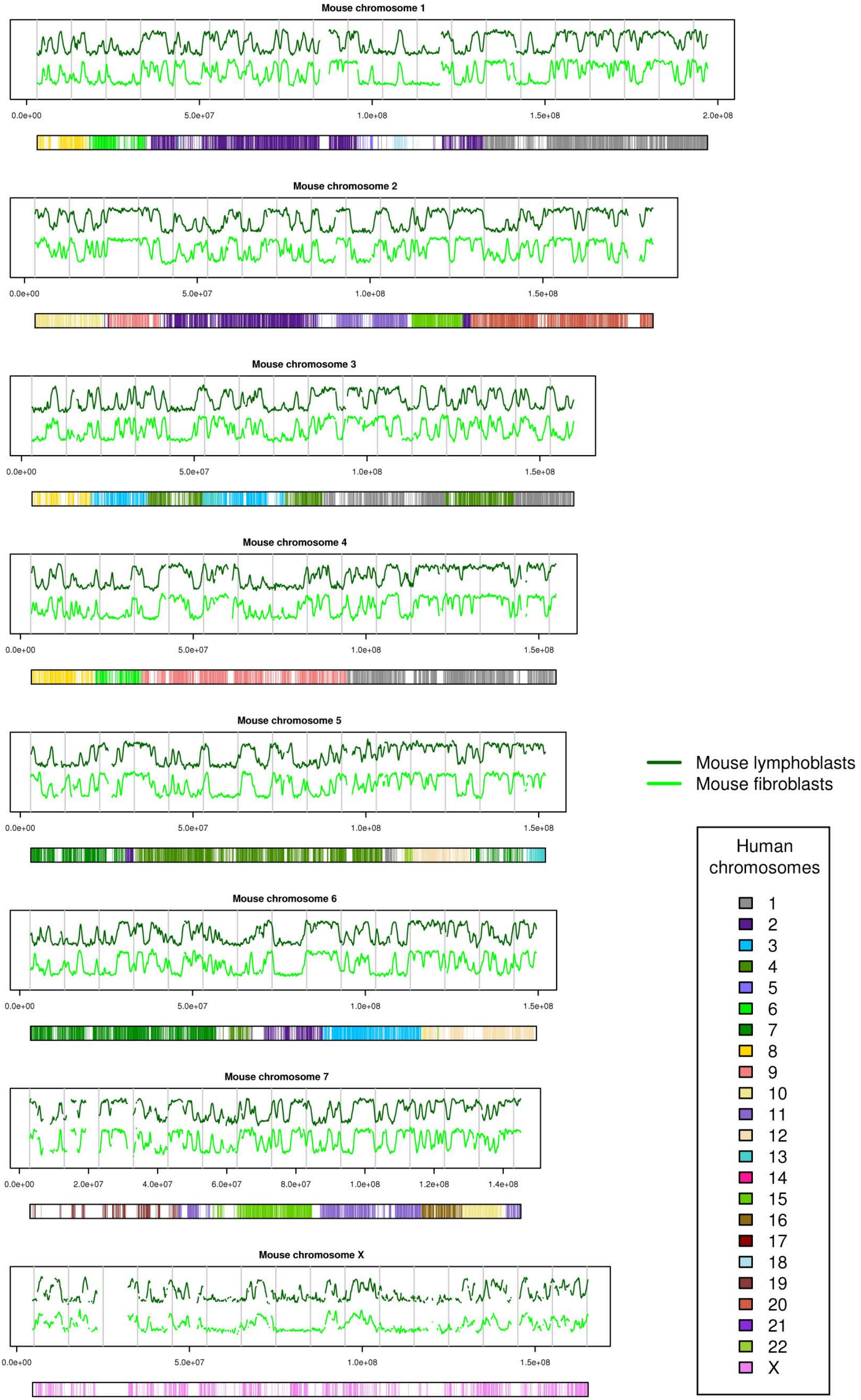
Mouse chromosome 1
0.0e+00
5.0e+07
1.0e+08
1.5e+08
2.0e+08
Mouse chromosome 2
0.0e+00
5.0e+07
1.0e+08
1.5e+08
Mouse chromosome 3
0.0e+00
5.0e+07
1.0e+08
1.5e+08
Mouse chromosome 4
0.0e+00
5.0e+07
1.0e+08
1.5e+08
Mouse chromosome 5
0.0e+00
5.0e+07
1.0e+08
1.5e+08
Mouse chromosome 6
0.0e+00
5.0e+07
1.0e+08
1.5e+08
Mouse chromosome 7
0.0e+00
2.0e+07
4.0e+07
6.0e+07
8.0e+07
1.0e+08
1.2e+08
1.4e+08
Mouse chromosome X
0.0e+00
5.0e+07
1.0e+08
1.5e+08
Mouse lymphoblasts
Mouse fibroblasts
Human chromosomes
1
2
3
4
5
6
7
8
9
10
11
12
13
14
15
16
17
18
19
20
21
22
X

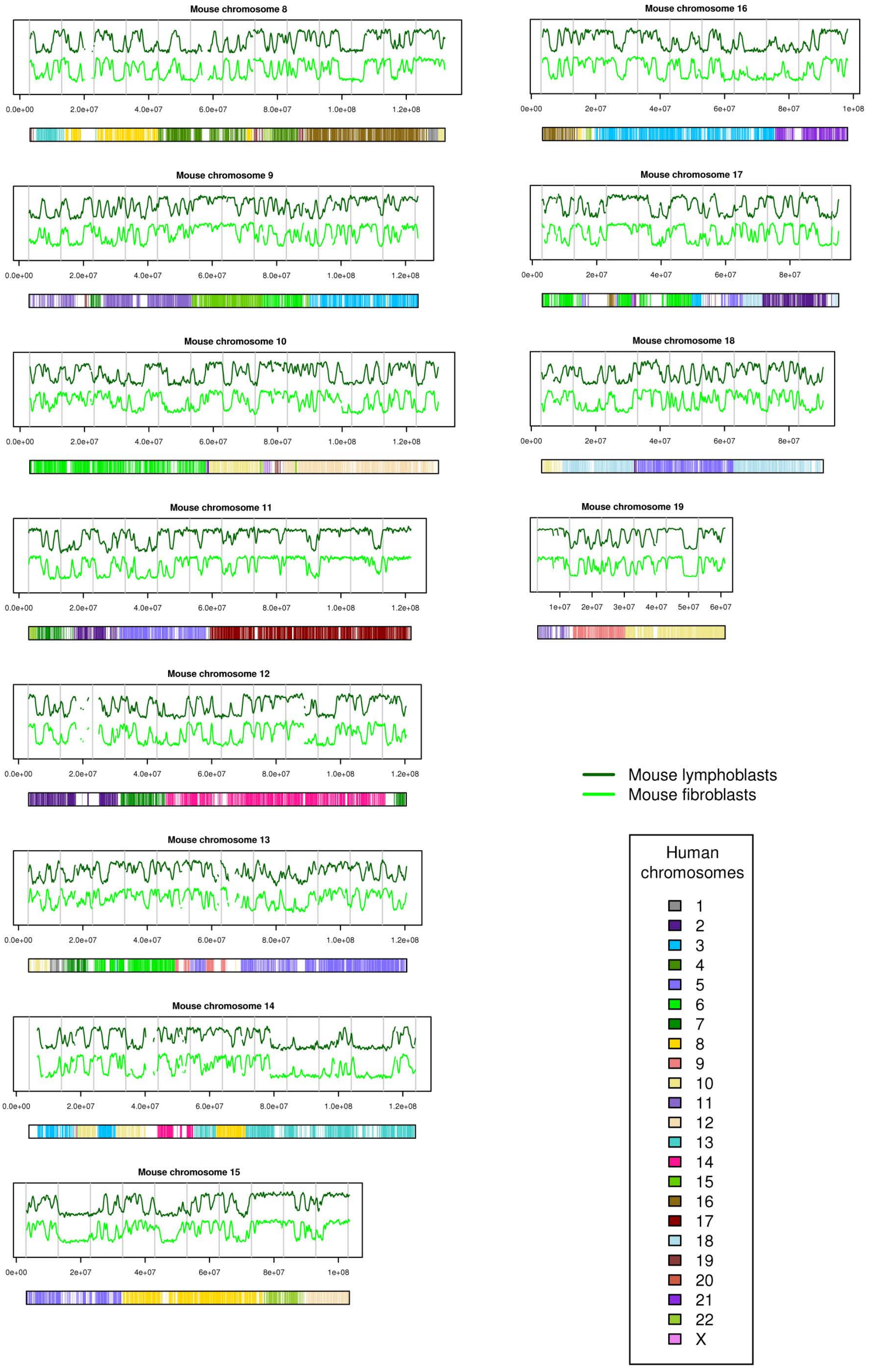
Mouse chromosome 8
0.0e+00 2.0e+07 4.0e+07 6.0e+07 8.0e+07 1.0e+08 1.2e+08
Mouse chromosome 9
0.0e+00 2.0e+07 4.0e+07 6.0e+07 8.0e+07 1.0e+08 1.2e+08
Mouse chromosome 10
0.0e+00 2.0e+07 4.0e+07 6.0e+07 8.0e+07 1.0e+08 1.2e+08
Mouse chromosome 11
0.0e+00 2.0e+07 4.0e+07 6.0e+07 8.0e+07 1.0e+08 1.2e+08
Mouse chromosome 12
0.0e+00 2.0e+07 4.0e+07 6.0e+07 8.0e+07 1.0e+08 1.2e+08
Mouse chromosome 13
0.0e+00 2.0e+07 4.0e+07 6.0e+07 8.0e+07 1.0e+08 1.2e+08
Mouse chromosome 14
0.0e+00 2.0e+07 4.0e+07 6.0e+07 8.0e+07 1.0e+08 1.2e+08
Mouse chromosome 15
0e+00 2e+07 4e+07 6e+07 8e+07 1e+08
Mouse chromosome 16
0e+00 2e+07 4e+07 6e+07 8e+07 1e+08
Mouse chromosome 17
0e+00 2e+07 4e+07 6e+07 8e+07
Mouse chromosome 18
0e+00 2e+07 4e+07 6e+07 8e+07
Mouse chromosome 19
1e+07 2e+07 3e+07 4e+07 5e+07 6e+07
Mouse lymphoblasts
Mouse fibroblasts
Human chromosomes
1
2
3
4
5
6
7
8
9
10
11
12
13
14
15
16
17
18
19
20
21
22
X
