# Supplementary material for: Comparative Analysis of DNA Replication Timing Reveals Conserved Large-Scale Chromosomal Architecture
Source: PLoS Genet. 2010 Jul 1;6(7):e1001011. doi: 10.1371/journal.pgen.1001011 (PMC2895651; doi:10.1371/journal.pgen.1001011)

Event 346, type:fusion, branch:(mouse,rat)

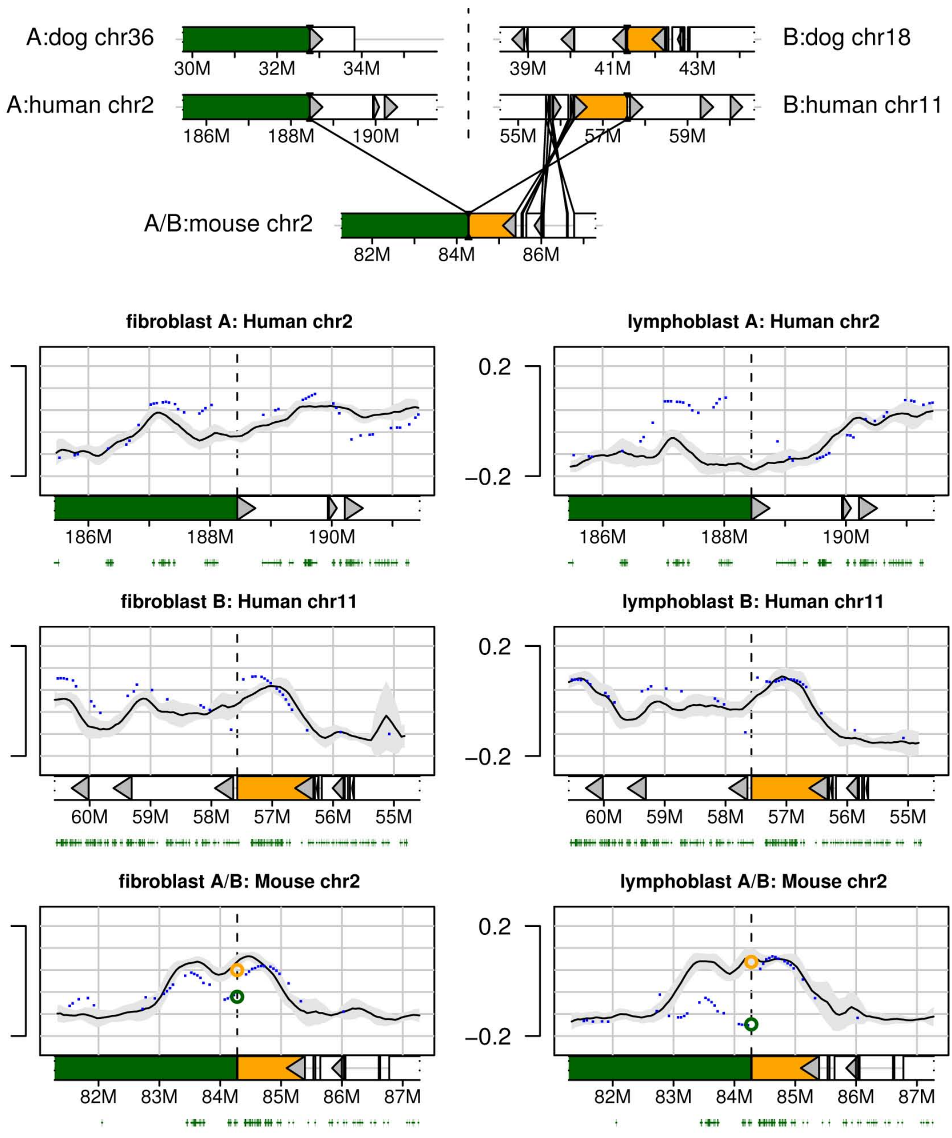

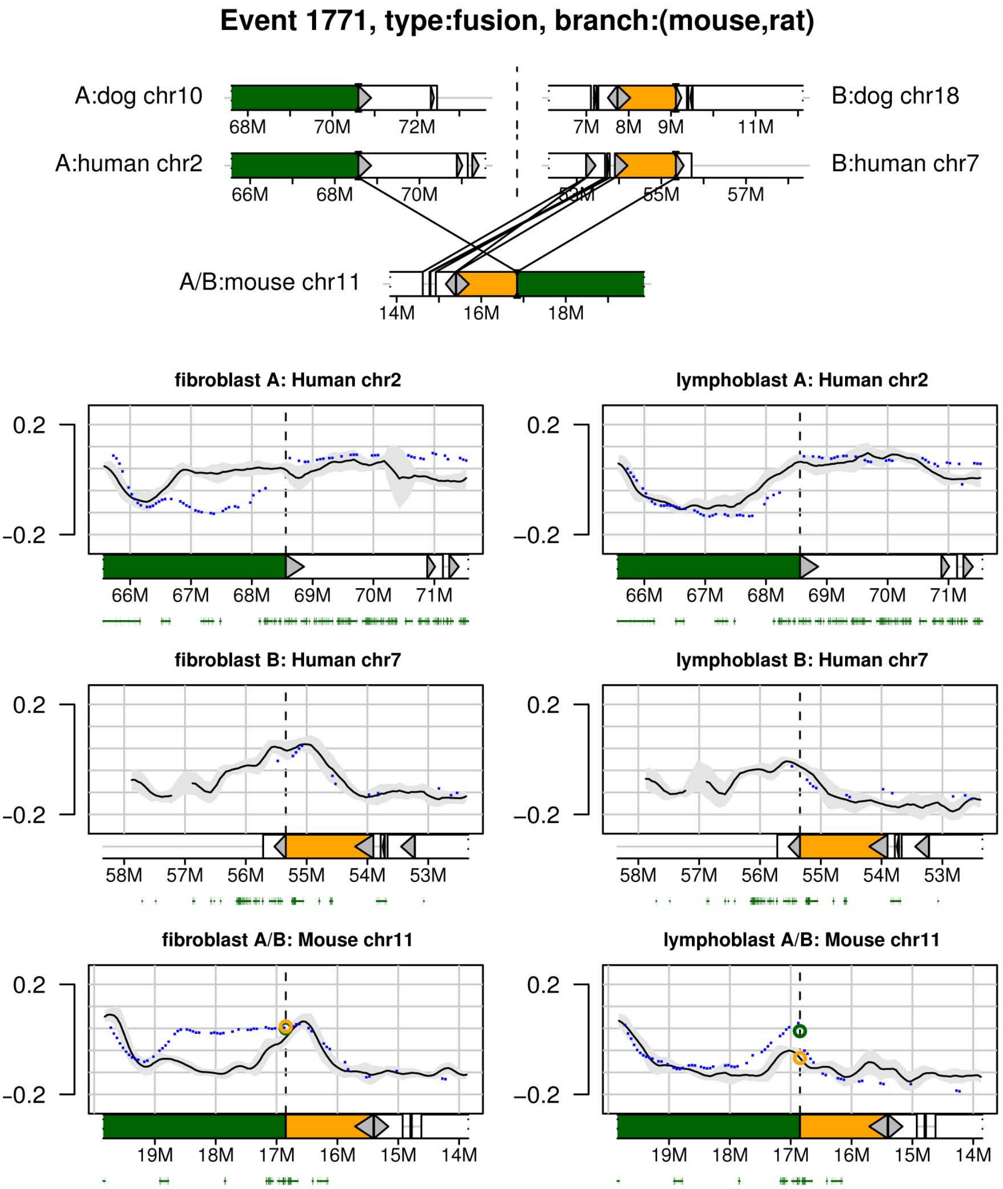
Event 1771, type:fusion, branch:(mouse,rat)
A:dog chr10
B:dog chr18
A:human chr2
B:human chr7
A/B:mouse chr11
fibroblast A: Human chr2
lymphoblast A: Human chr2
fibroblast B: Human chr7
lymphoblast B: Human chr7
fibroblast A/B: Mouse chr11
lymphoblast A/B: Mouse chr11

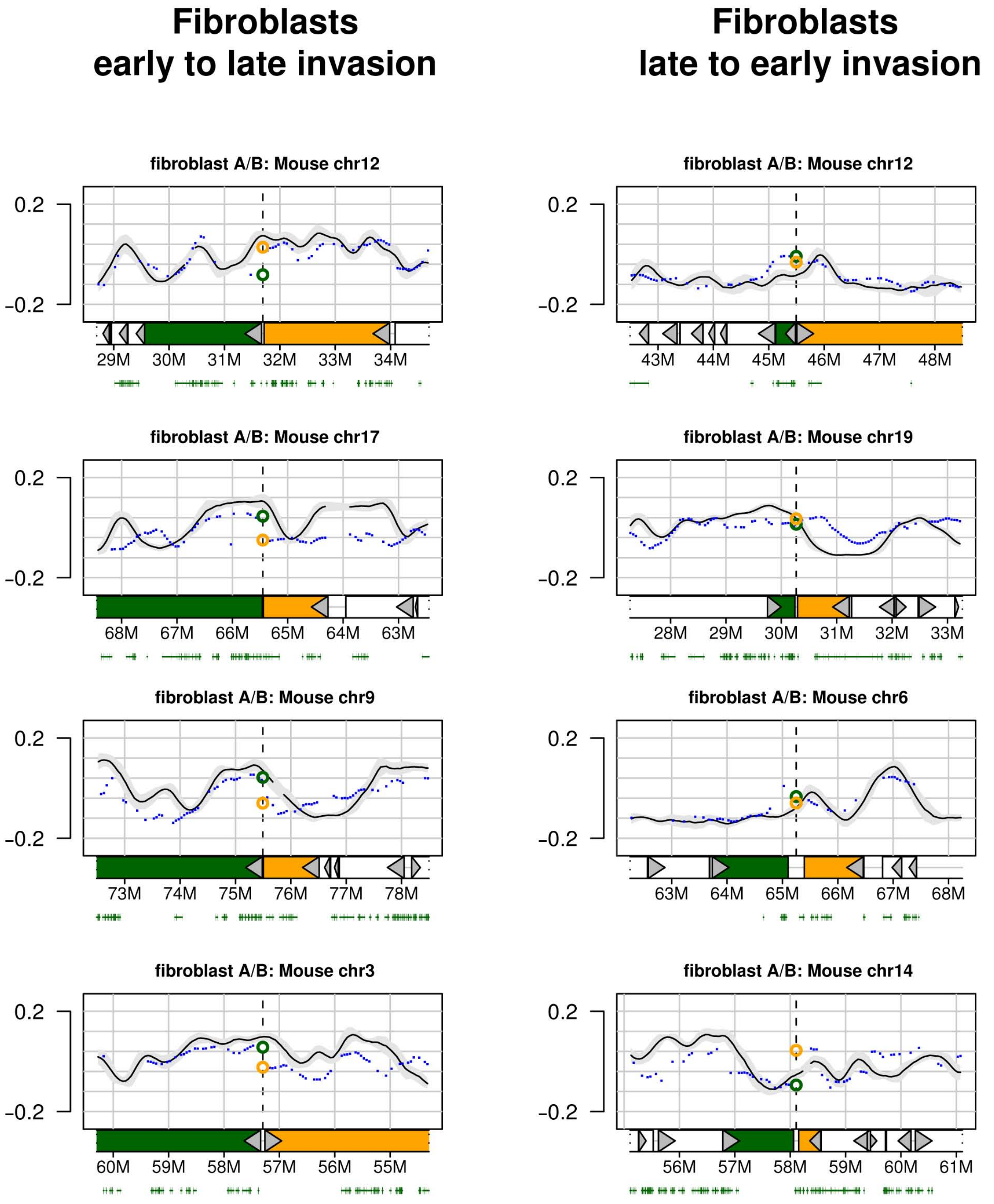
Fibroblasts
early to late invasion
Fibroblasts
late to early invasion
fibroblast A/B: Mouse chr12
0.2
-0.2
29M
30M
31M
32M
33M
34M
fibroblast A/B: Mouse chr12
43M
44M
45M
46M
47M
48M
fibroblast A/B: Mouse chr17
68M
67M
66M
65M
64M
63M
fibroblast A/B: Mouse chr19
28M
29M
30M
31M
32M
33M
fibroblast A/B: Mouse chr9
73M
74M
75M
76M
77M
78M
fibroblast A/B: Mouse chr6
63M
64M
65M
66M
67M
68M
fibroblast A/B: Mouse chr3
60M
59M
58M
57M
56M
55M
fibroblast A/B: Mouse chr14
56M
57M
58M
59M
60M
61M

Lymphoblasts early to late invasion

Lymphoblasts late to early invasion

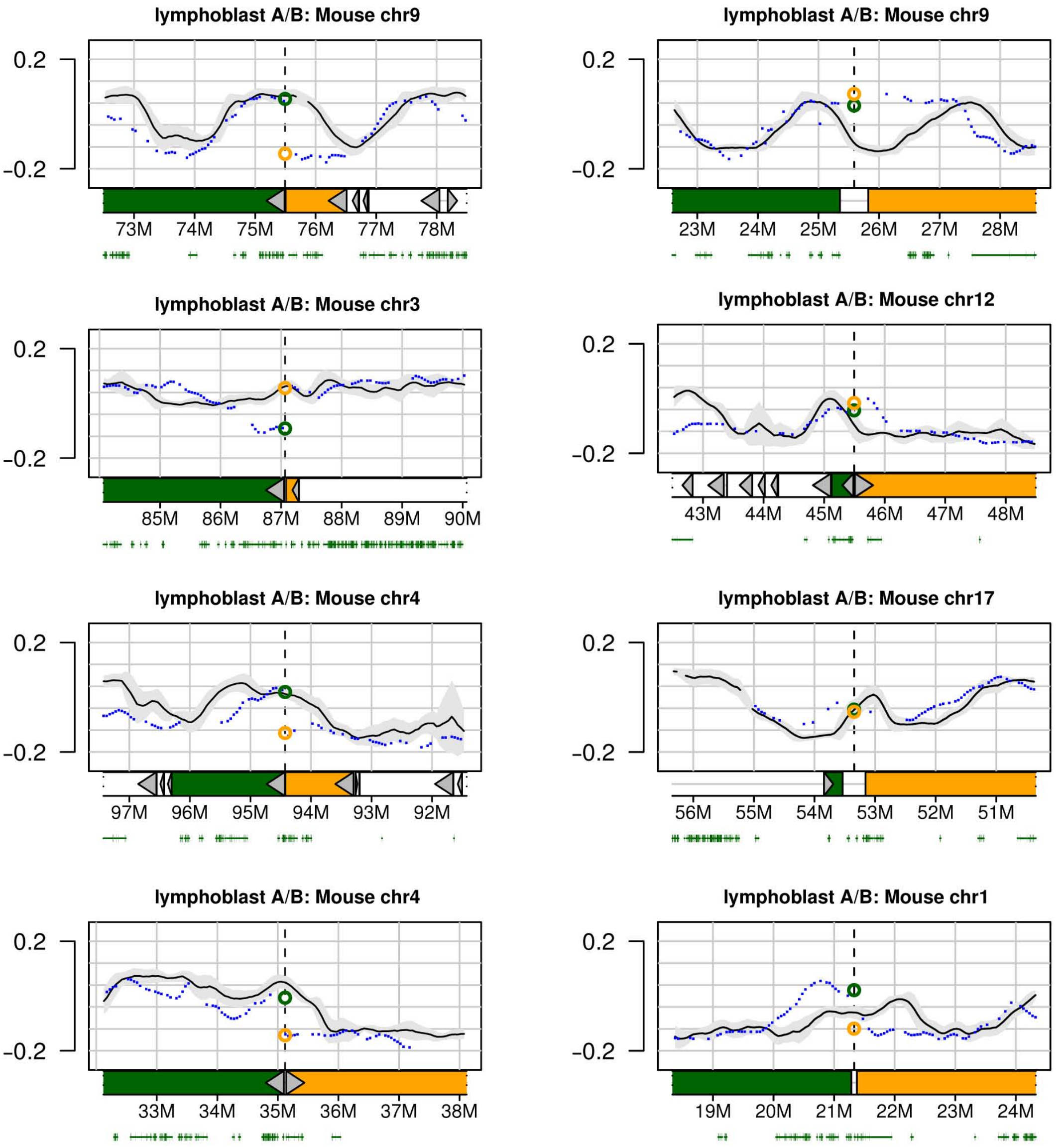

Supplement: Figure S12 — Fusion examples. ToR divergence near near murine fusion sites. Gene density is marked with green, under each plot. We depict ToR with a black line (the confidence interval is shown in grey), and depict projected ToR with blue dots. The two segments that got fused in the mouse lineage are colored green (left segment) and orange (right segment). The approximated ToR near the breakpoint prior to fusion is depicted with a colored circle (green and orange) for both segments. (A,B) are detailed versions of Figure 4D. In (C,D) we show for both celltypes the top diverged events of both early-to-late invasion (red in Figure 4E) and late-to-early invasion (blue in Figure 4E). (1.25 MB PDF) [file pgen.1001011.s012.pdf]
